# Exercise training counteracts urothelial carcinoma-induced alterations in skeletal muscle mitochondria phospholipidome in an animal model

**DOI:** 10.1038/s41598-019-49010-6

**Published:** 2019-09-17

**Authors:** Javier-Fernando Montero-Bullon, Tânia Melo, Rita Ferreira, Ana Isabel Padrão, Paula A. Oliveira, M. Rosário M. Domingues, Pedro Domingues

**Affiliations:** 1 Centro de Espectrometria de Massa, Departamento de Química & QOPNA, Universidade de Aveiro, Campus Universitário de Santiago, 3810-193 Aveiro, Portugal; 2 CIAFEL, Faculty of Sports, University of Porto, Porto, Portugal; 3 CITAB, Department of Veterinary Sciences, School of Agrarian and Veterinary Sciences, University of Trás-os-Montes and Alto Douro (UTAD), Vila Real, Portugal; 4 Departamento de Química & CESAM&ECOMARE, Universidade de Aveiro, Campus Universitário de Santiago, 3810-193 Aveiro, Portugal

**Keywords:** Lipidomics, Mass spectrometry

## Abstract

Cancer associated body wasting is the cause of physical disability, reduced tolerance to anticancer therapy and reduced survival of cancer patients and, similarly to cancer, its incidence is increasing. There is no cure for this clinical condition, and the pathophysiological process involved is largely unknown. Exercise training appears as the gold standard non-pharmacological therapy for the management of this wasting syndrome. Herein we used a lipidomics approach based on liquid chromatography coupled with high-resolution mass spectrometry (LC-HR-MS) to study the effect of exercise in the modulation of phospholipids profile of mitochondria isolated from *gastrocnemius* muscle of a pre-clinical model of urothelial carcinoma-related body wasting (BBN induced), submitted to 13 weeks of treadmill exercise after diagnosis. Multivariate analysis showed a close relationship between the BBN exercise group and both control groups (control sedentary and control exercise), while the BBN sedentary group was significantly separated from the control groups and the BBN exercise group. Univariate statistical analysis revealed differences mainly in phosphatidylserine (PS) and cardiolipin (CL), although some differences were also observed in phosphatidylinositol (PI, LPI) and phosphatidylcholine (PC) phospholipids. PS with shorter fatty acyl chains were up-regulated in the BBN sedentary group, while the other species of PS with longer FA and a higher degree of unsaturation were down-regulated, but the BBN exercise group was mostly similar to control groups. Remarkably, exercise training prevented these alterations and had a positive impact on the ability of mitochondria to produce ATP, restoring the healthy phospholipid profile. The remodelling of mitochondria phospholipid profile in rats with urothelial carcinoma allowed confirming the importance of the lipid metabolism in mitochondria dysfunction in cancer-induced skeletal muscle remodelling. The regulation of phospholipid biosynthetic pathways observed in the BBN exercise group supported the current perspective that exercise is an adequate therapeutic approach for the management of cancer-related muscle remodeling.

## Introduction

The incidence of cancer has increased in the last years and, consequently, paraneoplastic syndromes such as cachexia have also increased. Cachexia is an insidious syndrome associated with cancer, estimated to occur in 60–80% of the cases, and is mainly characterized by body weight decrease due to skeletal muscle loss^1,2^. This muscle wasting contributes to physical disability, weakness, reduced tolerance to anticancer therapy and reduced survival of cancer patients^1,2^. Mitochondrial dysfunction is an early molecular event reported in cancer-related skeletal muscle wasting^3–6^. ATP production has been shown to be impaired^7^, mainly due to the downregulation of oxidative phosphorylation (OXPHOS) complexes I, II, IV and V expression and activity^8,9^, and by energy dissipation through uncoupling proteins (UCP)^8–10^. Changes in mitochondrial phospholipids profile has been described and seem to be mainly characterized by the decrease of cardiolipin (CL), its precursor phosphatidylglycerol (PG) and phosphatidic acid (PA), and by the increase of phosphatidylcholine (PC) and phosphatidylserine (PS) contents^8^. CL is the only lipid that is synthesized in mitochondria^11^, where it performs a critical role in the organization of mammalian OXPHOS complexes and in the maintenance of inner mitochondrial membrane potential^11^. So, decreased levels of CL in mitochondria seem to explain, at least in part, the impaired ability of skeletal muscle from tumour-bearing animals to synthesize ATP^8^. Oxidative stress is also involved in cachexia and increased ROS cause oxidation of biomolecules, and could be one of the causes of decreased CL content and mitochondrial dysfunction^8,12^.

Exercise training appears as the gold standard non-pharmacological therapy for increasing muscle function and counteracting the dramatic reduction of muscle strength and endurance that characterizes cancer cachexia^6,13–15^. The practice of exercise was shown to decrease the serum levels of pro-inflammatory cytokines^13,16,17^, to increase muscle protein synthesis and attenuate proteolysis^10^, to improve mitochondrial dynamics^17,18^ and modulate redox imbalance^19^. Indeed, the benefits of exercise training on skeletal muscle involves mitochondrial adaptations mainly characterized by increased mitochondrial biogenesis, which are linked to improved metabolic health^20^. A single bout of endurance exercise was shown to induce a rapid and sustained increase of PGC-1α, a central player in mitochondria biogenesis in skeletal muscle^21^. Moreover, PGC-1α seems to mediate exercise training-related increase of the enzymes involved in CL and PI synthesis^22^. However, the molecular events underpinning the benefits of exercise training in counteracting cancer-induced muscle wasting, particularly the ones harboured in mitochondria, remain elusive.

In the present study, we use a lipidomics MS-based approach to study the effect of exercise in the modulation of phospholipids profile of mitochondria isolated from *gastrocnemius* muscle of a pre-clinical model of urothelial carcinoma-related body wasting submitted to 13 weeks of treadmill exercise after diagnosis. The identification of changes in the phospholipid profile permits to hypothesize about the physiological processes involved, and possible biomarkers and therapeutic targets of cancer-related muscle remodelling and physical exercise beneficial role.

## Material and Methods

### Chemicals

*N-butyl-N-(4-hydroxybutyl)-nitrosamine* (BBN) was purchased from Tokyo Kasey Kogyo (Japan). Rabbit polyclonal anti-PGC-1α (ab54481) and secondary peroxidase-conjugated (anti-rabbit IgG; ab ab6721) antibodies were purchased to Abcam (Cambridge, UK). Phospholipids internal standards used were (1,3-bis[1,2-dimyristoyl-sn-glycero-3-phospho]-sn-glycerol) (CL(14:0)_4_), 1,2-dimyristoyl-sn-glycero-3-phosphocholine (dMPC), 1-nonadecanoyl-2-hydroxy-sn-glycero-3-phosphocholine (LPC(19:0)), 1,2-dimyristoyl-sn-glycero-3-phosphoethanolamine (dMPE), 1,2-dimyristoyl-sn-glycero-3-phosphate (dMPA), 1,2-dimyristoyl-sn-glycero-3-phospho-(10-rac-glycerol) (dMPG), 1,2-dimyristoyl-sn-glycero-3-phospho-L-serine (dMPS), 1,2-dipalmitoyl-sn-glycero-3-phospho-(10-myo-inositol) (dPPI), N-heptadecanoyl-D-erythro-sphingosylphosphorylcholine (SM(17:0/d18:1)) and N-heptadecanoyl-D-erythro-sphingosine (Cer(17:0/d18:1)), purchased to Avanti polar lipids Inc (Alabaster, AL), as used in previous studies from our lab^23,24^. Organic solvents of HPLC grade (chloroform, methanol, acetonitrile) were purchased from Fisher Scientific (Leicestershire, UK). Other reagents such as ammonium acetate from Sigma-Aldrich (St. Louis, MO, USA), perchloric acid (HClO_4_, 70%) and ammonium molibdate tetrahydrate ((NH_4_)_6_Mo_7_O_24_·4H_2_O) from Panreac (Barcelona, Spain), ascorbic acid (C_6_H_8_O_6_) from VWR International (Leicestershire, UK), sodium dihydrogen phosphate dihydrate (NaH_2_PO4·2H_2_O) from Riedel-de Haёn (Seelze, Germany)were used as received. Milli-Q water was filtered using a a Milli-Q Millipore system (MilliQ plus 185).

### Animals and experimental design

Forty-four male Wistar rats (aged 5 weeks) were obtained from Harlan (Barcelona, Spain) and randomly housed in groups of 4 rats/cage, in a controlled environment at 22 ± 2 °C of temperature and 60 ± 5% of relative humidity with 12/12 hour dark-light inverted cycle, with free access to food (standard laboratory diet 4RF21®, Mucedola, Italy) and water. After one week of acclimatization, the animals were randomly divided into two experimental groups: exposed to 0.05% BBN in the drinking water over the course of 20 weeks (BBN group, n = 24) and with access to tap water (CT, n = 20). After this 20 week-period, half of the animals from each group started an exercise program in a treadmill running for 13 weeks (subgroups BBNex (n = 12) and CTex (n = 10)). The animal protocol was approved by the Portuguese Ethics Committee for Animal Experimentation, Direção Geral de Alimentação e Veterinária (license number 008962) and was performed in accordance with the European Directive 2010/63/EU. This protocol was previously reported to study cardiac remodelling^25^.

Animals from the EX group were submitted to a treadmill exercise training program on an electric treadmill (Treadmill Control® LE 8710, Panlab, Harvard Apparatus, ISA) for 13 weeks during 5 days/week. In the first two weeks, exercise duration and treadmill speed were gradually increased until it reached 60 min per day at 20 m per min, which was maintained for 11 weeks. At the end of the experimental protocol, all animals were weighed and sacrificed with ketamine/xylazine (Imalgen® and Rompun®, respectively). All noticeable tumours were counted and bladders were removed for histological analysis, as previously reported^25^. In brief, urinary bladders were fixed [4% (v/v) buffered paraformaldehyde] by diffusion during 24 h and subsequently dehydrated with graded ethanol and included in paraffin blocks. Urinary bladder lesions were classified histologically using haematoxylin and eosin-stained slides^26^. *Gastrocnemius* muscle was removed, weighed, and immediately prepared for mitochondria isolation.

### Mitochondria isolation from *gastrocnemius* muscle

Mitochondria isolation was performed using the conventional methods of differential centrifugation, as previously described^27^. All the procedures were performed on ice or below 4 °C. Briefly, muscles were immediately excised and minced in ice-cold isolation medium containing 100 mM sucrose, 0.1 mM ethylene glycol tetraacetic acid, 50 mM Tris–HCl, 100 mM KCl, 1 mM KH_2_PO_4_, and 0.2% free fatty acid bovine serum albumin (BSA), pH 7.4. Minced blood-free tissue was rinsed and suspended in 10 mL of fresh medium containing 0.2 mg/mL bacterial proteinase (Nagarse E.C.3.4.21.62, type XXVII; Sigma) and stirred for 2 min. The sample was then carefully homogenized with a tightly fitted Potter-Elvehjem homogenizer and a Teflon pestle. An aliquot of homogenized was separated for biochemical analysis. After homogenization, three volumes of Nagarse-free isolation medium were added to the homogenate, which was then centrifuged at 700 g for 10 min. The resulting supernatant suspension was centrifuged at 10,000 g for 10 min. The pellet was gently re-suspended in the isolation medium (1.3 mL/100 mg initial tissue) and centrifuged at 7000 g for 3 min. The final pellet, containing the mitochondrial fraction, was gently re-suspended (0.4 mL/mg initial tissue) in a medium containing 225 mM mannitol, 75 mM sucrose, 10 mM Tris, and 0.1 mM EDTA, pH 7.4. Mitochondrial protein content was determined using the Bio-Rad RC-DC assay.

### Immunoblotting analysis of PGC-1α

Equivalent amounts of whole *gastrocnemius* muscle protein of each group (30 µg) were electrophoresed on a 12.5% SDS-PAGE as described by Laemmli^28^. Gels were blotted onto a nitrocellulose membrane (Whatman®, Protan) in transfer buffer (25 mM Tris, 192 mM glycine, pH 8.3 and 20% methanol) during 2 hours (200 mA). Then, nonspecific binding was blocked with 5% (w/v) dry nonfat milk in TBS-T (100 mM Tris, 1.5 mM NaCl, pH 8.0 and 0.5% Tween 20). Membranes were incubated with primary antibody diluted 1:1000 in 5% (w/v) fat free milk in TBS-T (anti-PGC1α ab54481) overnight at 4 °C, washed and incubated with goat anti-rabbit IgG-HRP secondary antibody (ab6721), for 1 h at room temperature. Immunoreactive bands were detected by enhanced chemiluminescence ECL (Amersham Pharmacia Biotech) according to the manufacturer’s procedure and images were recorded using a Molecular Imager Gel Doc XR + System (Bio-Rad Laboratories) and analyzed with ImageLab software (version 5.0, Bio-Rad Laboratories). Protein loading control was performed with Ponceau S staining^29^.

### Determination of ATP synthase activity

The activity of ATP synthase was measured as previously described^30^. In brief, the phosphate produced by the hydrolysis of ATP reacts with ammonium molybdate, in the presence of reducing agents, forming a blue-colour complex. The colour intensity was proportional to the concentration of phosphate in solution. Optical densities were measured at 610 nm in a Multiskan GO Microplate Spectrophotometer (Thermo Scientific). Oligomycin was used as an inhibitor of mitochondrial ATPase activity.

### Determination of citrate synthase activity

Citrate synthase (CS) activity was measured in mitochondrial fractions using the method proposed by Coore, Denton^31^. In brief, the CoASH released from the reaction of acetyl-CoA with oxaloacetate was measured by its reaction with 5,5′-dithiobis-(2-nitrobenzoic acid) (DTNB) at 412 nm (ε = 13.6 mM^−1^cm^−1^). Total citrate synthase activity was expressed in nanomoles per milligrams of mitochondrial protein.

### Extraction of mitochondrial phospholipids

Lipid extraction of each mitochondrial fraction was performed according to the Bligh and Dyer method^32^. Briefly, 3.75 mL of chloroform/methanol 1:2 (v/v) was added to 1 mL of mitochondrial fraction (corresponding approximately to 8 mg of protein). Then, the tubes were well-vortexed and incubated on ice for 30 min (vortex every 5 min). An additional volume of 1.25 mL chloroform and 1.25 mL milli-Q H_2_O were added. Finally, following vigorous vortex, samples were centrifuged at 1000 rpm (Mixtasel centrifuge, Selecta), for 5 min at room temperature to obtain a two-phase system: aqueous top phase and organic bottom phase from which lipids were obtained. The organic phase was collected to a new tube, and the aqueous phase was washed with 1.88 mL of chloroform. At last, the extracts were dried in a nitrogen flow, dissolved in 1 mL of chloroform for subsequent quantification by the phosphorus assay.

### Quantification of phospholipids content by phosphorus assay

The total amount of phospholipids in each lipid extract was determined in two replicate experiments by Barlett and Lewis colourimetric assay, based on the measurement of inorganic phosphorus, as done in our laboratory^23,24^. Briefly, an aliquot of 10 µL of the extract was dried and incubated 1 h at 180 °C with 125 µL of perchloric acid (70%) in a heating block (Stuart, U.K.) to hydrolyse the inorganic phosphorus of the phospholipids. Afterwards, and once cooled down to room temperature, the solutions were mixed by vortexing with 825 µL of Milli-Q water, 125 µL of (NH_4_)_6_Mo_7_O_24·4_H_2_O) 2.5% and 125 µL of ascorbic acid 10% freshly prepared. Samples and standards (8 standards solutions, with different concentrations of phosphorus) were simultaneously incubated at 100 °C in a water bath for 10 min and cooled down. The content of inorganic acid was measured in a microplate spectrophotometer (Multiscan 90, ThermoScientific) at 797 nm.

### HILIC-ESI-MS and MS/MS

An amount of lipid extract equivalent to 5 µg of total phospholipid in 5 µL of chloroform was mixed with 4 µL of the standards solution (0.02 µg dMPC, 0.02 µg dMPE, 0.04 µg dMPS, 0.012 µg dMPG, 0.08 µg dMPA, 0.08 µg dPPI, 0.02 µg SM(17:0/d18:1), 0.02 µg Cer(17:0/d18:1), 0.08 µg CL(14:0)_4_ and 0.02 µg LPC(19:0) and 91 µL of initial HPLC conditions solvent mixture. For the HPLC-MS analysis, 5 µL were injected in an Ultimate 3000 Dionex ultra high-performance LC (UHPLC) system (Thermo Fisher Scientific, Bremen, Germany) coupled to a Q-Exactive hybrid quadrupole mass spectrometer (Thermo Fisher, Scientific, Bremen, Germany)^24^. A microbore chromatographic column Ascentis Si HPLC column, 15 cm length × 1.0 mm internal diameter × 3 µm particle size(Sigma–Aldrich) was used for the separation. A biphasic gradient was used with solvents A (25% water/50% acetonitrile/25% methanol (v/v/v), 1 mM of ammonium acetate) and B (60% acetonitrile /40% methanol (v/v), 1 mM of ammonium acetate). Initial condition with 40% of mobile phase A was held isocratically for 8 min, followed by a linear increase to 60% of A within 7 min and an isocratic period of 5 min, and return to the initial condition in linear decrease during 5 min with final 10 min of equilibration. The flow rate through the column was established 40 µL/min and the temperature at 30 °C. The Q-Exactive Orbitrap was operated with a HESI source simultaneously in positive mode (3 kV) and negative mode (−2.7 kV), with capillary temperature 250 °C, and sheath gas flow 15U and auxiliary gas 5U. The MS spectra were acquired at high resolution (70,000), with the AGC target set to 10E^6^ and maximum injection time of 200 ms. MS^2^ spectra were acquired in a different run and separately for positive and negative mode, with a pool of all the replicates of each condition adjusted to the same concentration as each replicate. For MS^2^, a ten data-dependent MS/MS scans were repeated continuously throughout the experiments with a resolution of 17 500, AGC target of 1E^5^, maximum injection time of 50 ms, minimum intensity threshold of 1E^4^, dynamic exclusion of 60 s, APEX trigger of 10 to 30 s, and isolation window of 1 Da. The normalized collision energy was stepped^25,30,33^. Data acquisition was carried out using the Xcalibur data system (V3.3, Thermo Fisher Scientific, Waltham, MA, USA).

### Data analysis and statistics

Data from LC-MS and MS/MS was analysed with XCalibur Qual Browser (Thermo Fisher Scientific, Waltham, MA, USA) for chromatographic, high-accuracy MS and MS^2^ identification of species. LC-MS data was checked against a the theoretic list of species, with exact *m/z* values of species that were probable and with retention times adapted based on the internal standard for each PL class. The program MZmine 2.26^34^ was used for high-resolution identification (5ppm) including retention time alignment, and plotting the chromatograms of the identified species together with the quantification of the area under the curve. Data pre-processing including baseline correction, peak deconvolution, deisotoping, alignment, and gap-filling was applied. Data of the area of each species was exported to an Excel data spreadsheet (Excel, Microsoft, Redmond, WA) and normalized by the ratio against a selected internal standard. The internal standards were endogenously non-occurring PL species, and each standard is employed for normalization of all the species of its class. Multivariate and univariate analyses were performed using R version 3.5^35^ in Rstudio version 1.1.4^33^. Data were glog transformed and autoscaled using the R package Metaboanalyst^36^. Principal Component Analysis (PCA) was conducted for exploratory data analysis, with the R built-in function and ellipses were drawn using the R package ellipse^37^, assuming a multivariate normal distribution and a level of 0.95. Kruskal-Wallis test *f*ollowed by Dunn’s post-hoc comparisons were performed with the R built-in function. Heatmaps were created using the R package pheatmap^38^ using “Euclidean” as clustering distance, and “ward.D” as the clustering method. Finally, volcano plots of fold change vs significance for a pairwise comparison using Wilcoxon test were performed in R. All graphics and boxplots were created using the R package ggplot2^39^. Other R packages used for data management and graphics included plyr^40^, dplyr^41^, tidyr^42^ and ggrepel^43^. Anthropometric, enzyme activities and protein content data are presented as mean ± standard deviation and were analyzed using Graph Pad Prism Statistical Software (version 5.0). D’Agostino & Pearson test was performed to check the normality of the data. The statistical significance of the differences between the experimental groups was determined using a two-way analysis of variance (ANOVA) followed by the Tukey multiple comparisons post hoc test. Results were considered significantly different when p < 0.05.

## Results

The phospholipidome of the total lipid extracts obtained from mitochondria of control rats (CTsed), healthy rats undertaking exercise (CTex), rats with urothelial carcinoma (BBNsed), and rats with urothelial carcinoma and submitted to exercise training (BBNex), were analysed by HILIC-MS and MS/MS and statistic analysis. The animal protocol was the same previously reported for the study of heart remodelling^25^.Thus, due to the descriptive nature of the data, the body and *gastrocnemius* weights shown in Table 1 were already presented^25^.

**Table 1:** Characterization of the animals‘ response to the BBN-induced muscle wasting and/or exercise training in terms of body weight, *gastrocnemius* mass and of the ratio *gastrocnemius*-to-body weight and mitochondrial ATP synthase activity.

	Experimental groups			
	CTsed	BBNsed	CTex	BBNex
body weight (g)	484.80 ± 31.90	440.66 ± 22.44*	502.70 ± 11.87	426.12 ± 33.51^¥¥¥^
*gastrocnemius* mass (g)	4.92 ± 0.46	4.66 ± 0.31	5.18 ± 0.53	4.86 ± 0.41
*gastrocnemius*-to-body weight (mg/g)	10.15 ± 0.68	10.57 ± 0.55	10.32 ± 1.20	11.42 ± 0.61^¥^

Values are expressed as mean ± standard deviation. Data previously presented^25^.

*p < 0.05 vs CTsed; **p < 0.01 vs CTsed; ^¥^*p* < 0.05 vs CTex; ^¥¥¥^*p* < 0.001 vs CTex.

Data obtained from bladder histological analysis confirmed that animals from BBN groups developed urothelial lesions whereas animals from control groups did not develop any lesion. BBN-exposed animals with activity confined to the cage’s space presented more aggressive lesions in the bladder and inflammation compared to exercised ones^25^. Morover, tumor-bearing animals evidenced significantly lower body weight (p < 0.05 vs CTsed; Table 1), suggestive of cachexia. Trained BBN rats also evidenced a 15% reduction of body mass (p < 0.001 vs. CTex; Table 1). No significant effect of BBN exposure was noticed on *gastrocnemius* mass; however, an approximately 10% increase of the ratio between *gastrocnemius* mass to body weight was noticed in BBNex group (p < 0.05 vs CTex; Table 1).

**Figure 1:**
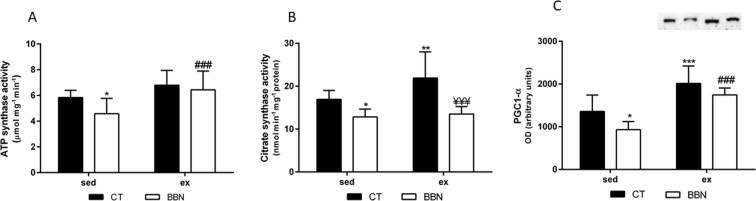
Effect of BBN exposure and/or exercise training on the mitochondrial activity of ATP synthase (**A**) and citrate synthase (**B**) and on the levels of PGC1-α in whole muscle homogenate (**C**). A representative immunoblot is shown above the correspondent graph (**C**; sample order has correspondence to the order of the groups presented in the graph). Values are expressed as mean ± standard deviation (*p < 0.05 vs CTsed; **p < 0.01 vs CTsed; ^¥¥¥^*p* < 0.001 vs CTex; ^###^p < 0.001 vs. BBNsed). Total Uncropped western blots for PGC1-alfa with the specific band highlighted (105 kDa; red arrow) are shown in Supplementary Fig. S1.

The effect of BBN exposure was noticed in the oxidative metabolic activity, characterized by a reduced ability of *gastrocnemius* muscle to produce ATP (Fig. 1A). Unlike the luciferase assay^44^, the one used in the present study measures ATP hydrolysis, which is the reverse mode of the ATP synthase. Indeed, tumor-bearing animals showed a significant decrease in CS and ATP synthase activities (*p* < 0.05 and *p* < 0.01 vs CTsed,). Exercise training counteracted the BBN effect on ATP synthase activity (*p* < 0.001 vs BBNsed). The effect of exercise training was corroborated by the significant higher activity of CS in the *gastrocnemius* of CT animals (*p* < 0.01 vs CTsed). Moreover, higher levels of PGC1α were observed in trained rats (from CT and BBN groups), suggestive of increased mitochondrial biogenesis (Fig. 1).

Using high resolution LC-MS, mass accuracy and by interpretation of LC-MS/MS data we identified and semi-quantified 211 molecular species of 12 lipid classes: PC, LPC, SM, PE, LPE, Cer, PG, PI, LPI, PA, PS, and CL (Supplementary Table S1). The identification was made using as criteria the *m/z* value, the retention time and manual analysis of the MS/MS spectra (Supplementary Table S1, Figs S2–S13). The molecular species of the classes PC, LPC, SM, PE, LPE, Cer were identified as [M + H]^+^ ions while PG, PI, LPI, PA, and PS, were identified as [M-H]^−^ ions. CL was identified as [M-2H]^2−^ ions.

**Figure 2:**
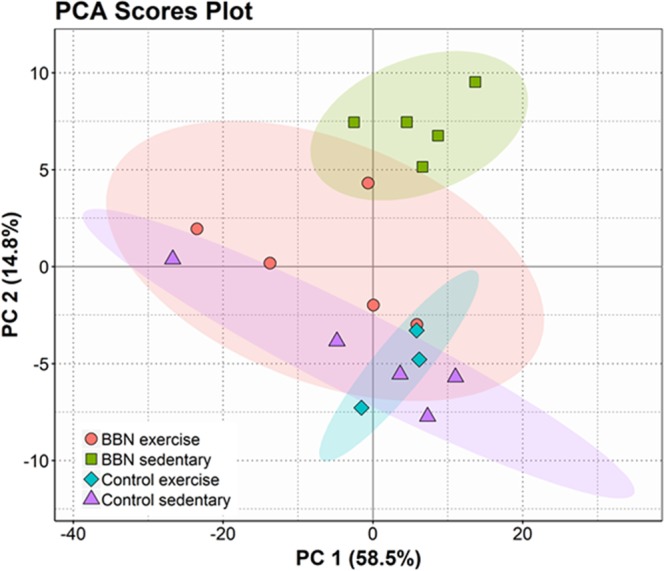
PCA score plot of the of the first two PCs of phospholipid data set acquired by LC-MS, of the four biological groups: Control sedentary, Control with exercise, urothelial cancer (BBN sedentary) and urothelial cancer submitted to exercise (BBN exercise).

The phospholipid profiles were then compared between the four conditions using univariate and multivariate analysis. To reduce the dimensionality of the data and visualize sample grouping, we performed principal component analysis (PCA) on the phospholipidomics data set. The visual observation of the PCA bi-plot for principal components 1 and 2 identified two outliers from the control exercise group (data not shown), which were removed from further analysis. The principal component analysis showed that the eigenvalues of the two first principal components represented 73.3% of the total variance (PC1 58.5%; PC2 14.8%) of the observations (Fig. 2). Figure 2 also shows significant segregation of the four cohorts along the second dimension, which is related to the variability of the distributions, whereas the first dimension is influenced by the values of the means within cohorts. The PCA of lipid profiles showed a close relationship between the BBNex, CTsed and CTex groups, while the BBNsed group was significantly separated from the CTsed and CTex groups. Remarkably, these results show that BBNex group is much more related to the CTsed group that of the BBNsed group.

**Figure 3:**
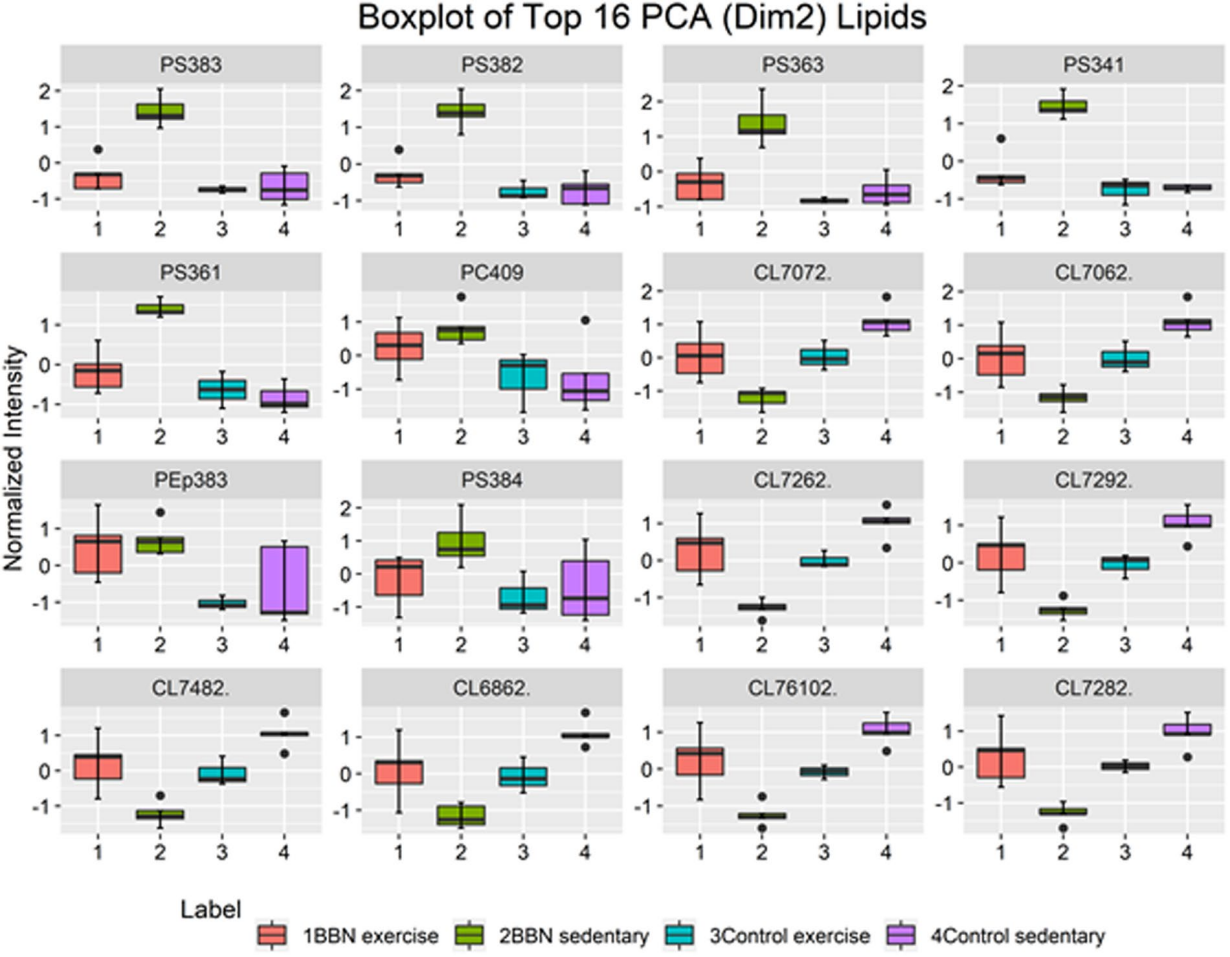
Boxplots of the 16 major phospholipid contributors of component 2 of the PCA. Labels of the species are according to the following notation: AAxxic (AA = lipid class; xx = total of carbon atoms in fatty acid; i = number of unsaturations; c = charge(cardiolipins).

We ranked the estimated coefficients (loadings) of component 2 of the PCA and selected the major 16 contributors. These species are shown in the box plots in Fig. 3. As it can be observed, the main contributors belong to PS (6 species) and CL (8 species). Figure 3 shows that a lower relative abundance of several CL species, independently of the number of carbons and unsaturations, occurs in the sedentary disease group (BBNsed). However, mitochondria from trained tumor-bearing rats (BBNex) presented relative abundances of CL at the levels of the control groups (CTex and CTsed). The PS content was found to be increased in the BBNsed group when compared with the CTsed, CTex and BBNex groups. Species as PC(40:9) and the plasmenyl PEp(38:3) were also important species, both of them showing a tendency to have lower relative abundance in the control groups.

**Figure 4:**
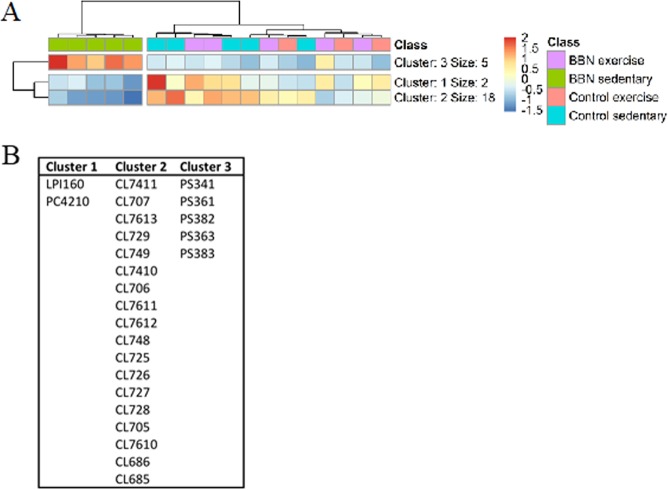
Two-dimensional hierarchical clustering heat map of the phospholipid data of the four studied groups. Levels of relative abundance are shown on the colour scale, with numbers indicating the fold difference from the mean (Fig. 4A). The clustering of the sample groups is represented by the dendrogram in the top, showing a cluster for BBNSed group, and another one for the remaining groups. The clustering of individual phospholipid species with respect to their similarity in changes of relative abundance is represented by the dendrogram to the left, showing 3 clusters. The members of each of these clusters are listed in Fig. 4B. Labels of the species are according to the following notation: AAxxic (AA = lipid class; xx = total of carbon atoms in fatty acid; i = number of unsaturations.

We performed an univariate analysis (Kruskal-Wallis and the post hoc *Dunn’s* multiple comparisons tests) of the various phospholipid species on the transformed dataset to test the association of these variables with the four clusters. This analysis allowed the identification of 45 species with p < 0.05, although after FDR correction, all had a p > 0.05 (Supplementary Table S2). Nevertheless, we have used the information from the univariate analysis to create a dendrogram with a two-dimensional hierarchical clustering, using the top 25 p-values phospholipid species (p < 0.02) (Fig. 4A). The primary split in the upper hierarchical dendrogram shows that the samples clustered independently in two groups: one cluster for BBNSed group, and another one for the remaining groups. The clustering of individual phospholipids with respect to their similarity in changes in phospholipid expression shows that they cluster in three groups. The first cluster of five PS species is clearly upregulated in disease BBNsed (cluster 3, Fig. 4B). A second group contains eighteen CL species that are decreased in the BBNSed group (cluster 2, Fig. 4B). And a third cluster includes LPI (16:0) and PC (42:10) also downregulated in the BBNSed group (cluster 1, Fig. 4B).

**Figure 5:**
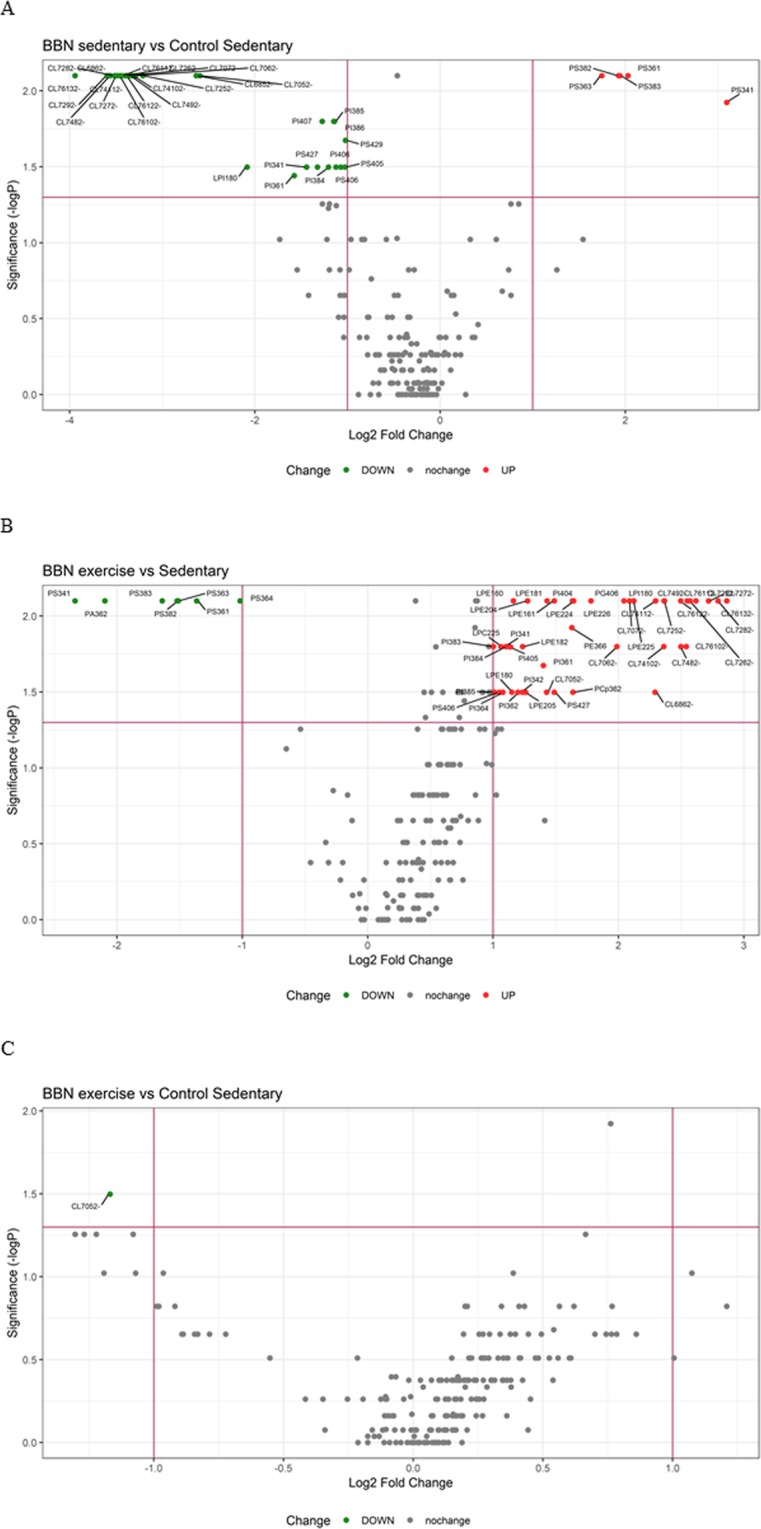
Volcano plot of all pairwise comparisons. Comparisons of all phospholipids from (**A**) BBN sedentary (*n* = 5) vs control sedentary (*n* = 5); (**B**) BBN sedentary (*n* = 5) vs BBN exercise (*n* = 5) and (**C**) BBN exercise (*n* = 5) vs control sedentary (*n* = 5). The volcano plot displays the relationship between fold-change and significance between the two groups. Significant phospholipids were selected by fold change (>2- or <−2-fold) and adjusted *Mann-Whitney* p-value (<0.05). Each dot denotes a phospholipid. The dashed red line *shows* where p = 0.05 and fold change = 2. Phospholipids identified as significant are coloured in red and labelled on the plot. Labels of the species are according to the following notation: AAxxic (AA = lipid class; xx = total of carbon atoms in fatty acid; i = number of unsaturations; c = charge(cardiolipins).

To conclude exploring the underlying phospholipid variations in the studied conditions, we undertook a pairwise phospholipid expression profiling analysis. The volcano plot displays significantly expressed phospholipid abundances against fold-change (log2 (ratio)) and p-value (−log10 (p-value), based on *Mann-Whitney* statistics for two treatments) (Fig. 5). The volcano plot in Fig. 5A showed the significant upregulated and downregulated differentially expressed phospholipids of the BBNsed versus the sedentary control group (CTsed). The volcano plot-based method revealed 35 differentially expressed phospholipids, of which 5 were upregulated in the BBNsed group, and 30 were downregulated. Downregulation was observed as the prominent regulation in tumor-bearing sedentary rats (BBNsed). The downregulated phospholipids comprised two different groups: Cardiolipins, with higher fold changes and lower p-values, and a different group with lower fold changes and higher p-values containing PS (4 species), PI (7 species) and LPI (1 species). Comparing with the sedentary control rats (CTsed), five PS were up-regulated in the sedentary rats that suffered from cachexia (BBNsed)((Supplementary Table S3). These PS contained shorter fatty acids (one C34, two C36 and two C38) than those that were upregulated (PS with C40 and C42).

The volcano plot in Fig. 5B showed the significant upregulated and downregulated differentially expressed phospholipids of the BBNex versus the BBNsed groups. The volcano plot revealed 51 differentially expressed phospholipids, of which 44 were upregulated in the BBNsed group, and 7 were downregulated. The downregulated phospholipids comprised 6 PS with shorter chain fatty acid (four C36 and two C38) and one PA. Of the upregulated group, cardiolipins (17species), with lower fold changes and lower p-values were the most significant feature. Other up-regulated species included PI (10 species), LPE (10 species) PS (2 species), LPI (1 species), PG (1 species), PCp (1 species), PE (1 species), LPC (1 species). The volcano plot in Fig. 5C showed the significant upregulated and downregulated differentially expressed phospholipids of the BBNex vs CTsed group. Remarkably, only one differential expressed phospholipid was observed, CL70:5, which was downregulated (Supplementary Table S3).

Determination of phospholipid class relative amount showed a higher relative content in PC class in BBNsed group, a lower amount in CL, PE and PI classes in BBNsed (Supplementary table S4).

## Discussion

Body wasting is a major contributor to the high morbidity and mortality in cancer, but the molecular mechanism responsible for this condition remains unclear. Nevertheless, it is hypothesized that changes in mitochondrial functionality drive skeletal remodelling in cancer. Oxidative stress and alterations of the mitochondrial phospholipid composition has been strongly correlated to mitochondria malfunction^45^. To date, very few works addressed the relation between phospholipid composition and cancer-related muscle remodelling^46–48^ and only one was performed in mitochondria, reporting the variation of the relative content of few phospholipid classes related to skeletal muscle mitochondrial dysfunction in a rat model of urothelial carcinoma^8^. To the best of our knowledge, there are no studies that characterized the impact of exercise training on urothelial carcinoma-induced alterations in skeletal muscle mitochondrial lipidome.

In the present study, we report a comprehensive analysis of the variation of the mitochondrial phospholipidome of the skeletal muscle from rats with urothelial carcinoma, using high-resolution LC-MS, aiming to evaluate the beneficial effect of the exercise in cachexia. Multivariate principal component analysis (PCA) together with hierarchical cluster analysis (HCA) were used for data visualization and analysis. PCA revealed that CTsed, CTex and BBNex clustered in one group, but the BBNsed group clustered distinctly (Fig. 2). In the PCA, the BBNex group clustered closely to both control groups, revealing a lipidic chemo-phenotype more closely related to the non-pathological conditions. This tendency supported that exercise has modulatory effects in the phospholipid profile and could interfere with some of the molecular mechanisms involved in the development of cachexia. The 16 molecular species that mostly contributed to the PCA discrimination between conditions (Fig. 3) included 6 PS with shorter fatty acyl chains and low degree of unsaturation, that were up-regulated in BBNsed, and 8 CL that were down-regulated in BBNsed. One PC and one pPE species were down-regulated in both BBN conditions.

We have selected the 25 more discriminant species, showing smaller p values in the univariate analysis, to create a dendrogram (Fig. 4). The hierarchical clustering showed that in the primary dendrogram, there was noteworthy discrimination between BBNsed and the other groups, as previously shown in the PCA analysis. The secondary dendrogram produced 3 nodes with different composition in phospholipids; the first one included one LPI and one PC with long FA and unsaturated chains; the second was composed by CL species and the third with PS species with shorter FA. This also corroborates the tendency presented in the PCA, showing a closer phenotype of BBNex with CT groups, and the benefits of exercise in modulating the PL profile, particularly in PS and CL species.

To date, there is no effective pharmacological therapeutic approach to treat cancer-related body wasting; however, exercise training is known to promote benefit systemic and muscle adaptations, overcoming muscle maladaptive adaptations^6,14,49–53^. The results from our work showed that exercise training promoted a positive remodelling of the mitochondrial phospholipidome of the skeletal muscle from rats with urothelial carcinoma, evidenced by the increased ability to produce ATP, which seems to be associated with the regulation of phospholipid metabolism. Indeed, exercise training of rats with urothelial carcinoma counteracted the down-regulation of major CL species and the up-regulation of PS (Fig. 5A,C). Also, comparing BBNsed and BBNex (Fig. 5B), we can see that there is an up-regulation of CL and PI species and a down-regulation of PS species in the exercised BBNex animals, corroborating the positive effect of exercise training in the regulation of PL profile in mitochondria in order to restore the healthier one. Altogether, these results also suggest that the modification of the phospholipid metabolism can be used to classify cancer cachexia since there is distinct mitochondrial phospholipidome phenotype for this group.

Cardiolipin is a dimeric phospholipid found almost exclusively in mitochondria and play essential roles in modulating the properties and the homeostasis of the membrane structure^54^. Also, this is a very important lipid class in the regulation of the activity of several mitochondria proteins, particularly oxidative phosphorylation (OXPHOS) complexes, and bioenergetics related proteins^55^. The decrease of CL content and disturbance of the CL profile was reported as an early event in apoptosis and to be present in several pathologies such as Barth syndrome^56^ and others. Cardiolipin peroxidation is enhanced under oxidative stress condition and could be one of the main responsible for the decrease of CL and mitochondrial dysfunction and cell death^57^. In cancer, a decrease of mitochondria CL was found to be accompanied by a decrease in the expression of cytochrome c, and an increase of the ratio Bax/Bcl-2, and was correlated to OXPHOS dysfunction and consequently to muscle catabolism associated with cancer^8^. In the present study, we observed that the reduced ability of skeletal muscle mitochondria to produce ATP, was reverted by exercise (Table 1). Based on these results, we hypothesized that the decrease of mitochondrial ATP production is related to a decrease in the cardiolipin content, which negatively affects the mitochondrial function.

Alterations of PS profile in mitochondria have been scarcely addressed, and its effect in the mitochondrial function is still unknown. There is some evidence that PS is important for the mitochondria regulation, due to its role as a precursor of PE biosynthesis. PS is usually transported from the endoplasmatic reticulum to the mitochondria via mitochondria-associated membranes (MAM)^22^ and is converted to PE in a reaction catalysed by the inner mitochondrial enzyme phosphatidylserine decarboxylase (PSD)^58^. The observed increase of PS in BBNsed could be due to impaired conversion of PS to PE. In fact, the lack of PS decarboxylase in PSD knockout mice was correlated with mitochondrial defects and dysfunction^59^. However, in this study, only very few PE species were down-regulated in BBNsed, most probably because the other PE biosynthetic routes in mitochondria also regulate the PE levels. Interestingly, we saw an increase of LPE species when BBNex is compared with BBNsed (Fig. 5B). LPE is a lysophospholipid recognized as an intercellular signalling molecule, but its function is far from being completely known^60^. LPE mobilizes intracellular Ca^2+^ through G-protein-coupled receptor (GPCR) in some cells types and was recently reported to exhibit anti-apoptotic activity in PC-12 cells^61^. Very recently, it was reported that treatment of PISD patients, a mitochondrial disease gene encoding phosphatidylserine decarboxylase proenzyme, causing skeletal dysplasia, with lyso-PE allowed to restore mitochondrial morphology. In PISD, there is an impairment of the conversion of PS to PE and the administration of LPE seemed to increase the PE mitochondrial pool, restoring the normal mitochondrial function. Likewise, the increase of LPE with exercise, observed in this study, could be a factor contributing to balance the PE biosynthesis and reverting the mitochondrial dysfunction associated with cachexia.

In this work, a decrease of PI species in the BBNsed group was also observed, but its metabolic role in mitochondria is still unknown. However, PI seems to be correlated with the production of phosphatidylinositol (3,4,5)-trisphosphate (PIP3) and with signalling events^55^. Therefore, it is expected that a change on the PI homeostasis impacts negatively on the mitochondrial function.

Skeletal muscle mitochondria have a unique phospholipid composition that is highly dynamic and can adapt to face different energetic demands. It was reported that the PL profile is adapted to diet and exercise training^22^. Upon moderate intensity exercise there was an upregulation of CL production, probably to face the high demand for energy^22^. Treadmill exercise training in rats appears to increase mitochondrial PC without affecting PE, CL or PI relative abundances^62,63^. In our work, no major alterations were observed between CTsed and CTex rats, but additional studies on exercise-induced adaptation of mitochondrial phospholipids are needed, taking in consideration the type, intensity and duration of training programs. However, our results provide strong molecular evidence of the mitochondrial phospholipidome remodelling promoted by exercise training on the set of cancer-related muscle remodelling.

## Conclusions

The modification of the mitochondria phospholipidome was observed in the skeletal muscle in response to urothelial carcinoma, which could be correlated to mitochondria dysfunction. The observed phospholipid signature on this group was mainly characterized by a decrease of the content of cardiolipin and phosphatidylinositol, and an increased content of phosphatidylserine. Exercise training prevented these alterations and had a positive impact on the ability of mitochondria to produce ATP. While exercise had a limited impact in the phospholipidome in control rats, exercise promoted a significant modification in tumor-bearing rats, restoring the healthy phospholipid profile.

## Supplementary information

Supplementary Information
