# Supplementary material for: Exercise training counteracts urothelial carcinoma-induced alterations in skeletal muscle mitochondria phospholipidome in an animal model
Source: Sci Rep. 2019 Sep 17;9:13423. doi: 10.1038/s41598-019-49010-6 (PMC6748971; doi:10.1038/s41598-019-49010-6)
Supplement: Supplementary file 1 — Supplementary Information [file 41598_2019_49010_MOESM1_ESM.docx]

Supplementary Information

Exercise training counteracts urothelial carcinoma-induced alterations in skeletal muscle mitochondria phospholipidome in an animal model

Javier-Fernando Montero-Bullon^1^, Tânia Melo^1,4^**,** Rita Ferreira^1^, Ana Isabel Padrão ^1,2^ , Paula A. Oliveira **^3^** M Rosário M Domingues^1,4*^, Pedro Domingues^1^

^1^Centro de Espectrometria de Massa, Departamento de Química & QOPNA, Universidade de Aveiro, Campus Universitário de Santiago, 3810-193 Aveiro, Portugal

^2^CIAFEL, Faculty of Sports, University of Porto, Porto, Portugal

3CITAB, Department of Veterinary Sciences, School of Agrarian and Veterinary Sciences, University of Trás-os-Montes and Alto Douro (UTAD), Vila Real, Portugal

^4^Departamento de Química & CESAM&ECOMARE, Universidade de Aveiro, Campus Universitário de Santiago, 3810-193 Aveiro, Portugal

*Corresponding author: Rosário Domingues, Department of Chemistry, Campus Universitário Santiago, 3810-193 Aveiro, Portugal. Email: [mrd@ua.pt](mailto:mrd@ua.pt)

Supplementary Table S 1: Summary for LC-MS and MS/MS identification of the phospholipid molecular species that were quantified in the present study. Fatty acyl chain composition was deduced from the carboxylate ions observed in negative mode. In the cases in which a positive charge ion was chosen for the detection, the negative ion counterpart was checked to assign the fatty acyl chain composition. Exceptionally, sphingomyelins and ceramides fatty acyl composition was observed in positive mode based on the sphingoid base ions. Species are annotated as: PL(C:N), where PL is the phospholipid class, C the number of carbons and N the number of double bonds .Asterisk (*) in the fatty acyl column is added when there is not information about the fatty acyls chains (NA), but other reporter ions are observed.

| Species assigned | Ion | Exact m/z | Observed m/z | Error (ppm) | RT | Most probable fatty acyl chain content according to MS_2_ data |
| --- | --- | --- | --- | --- | --- | --- |
| Cer(d34:1) | [M+H]^+^ | 538.5194 | 538.5206 | 2.2 | 3.4 | (d18:1/16:0) |
| Cer(d36:1) | [M+H]^+^ | 566.5507 | 566.5521 | 2.5 | 3.4 | (d18:1/18:0) |
| Cer(d36:2) | [M+H]^+^ | 564.5350 | 564.5363 | 2.3 | 3.4 | - |
| Cer(d38:1) | [M+H]^+^ | 594.5820 | 594.5828 | 1.3 | 3.4 | (d18:1/20:0) |
| Cer(d38:2) | [M+H]^+^ | 592.5663 | 592.5678 | 2.5 | 3.4 | - |
| Cer(d40:1) | [M+H]^+^ | 622.6133 | 622.6140 | 1.1 | 3.4 | (d18:1/22:0) |
| Cer(d42:1) | [M+H]^+^ | 650.6446 | 650.6456 | 1.6 | 3.4 | (d18:1/24:0) |
| Cer(d42:2) | [M+H]^+^ | 648.6289 | 648.6300 | 1.7 | 3.4 | (d18:1/24:1) |
| CL(68:5) | [M-2H]^2-^ | 698.4710 | 698.4703 | -1.0 | 2.7 | (16:1/16:1/18:0/18:3) |
| CL(68:6) | [M-2H]^2-^ | 697.4632 | 697.4639 | 1.0 | 2.6 | (16:1/16:1/18:2/18:2) |
| CL(70:5) | [M-2H]^2-^ | 712.4867 | 712.4840 | -3.8 | 2.7 | - |
| CL(70:6) | [M-2H]^2-^ | 711.4788 | 711.4771 | -2.3 | 2.6 | (16:1/18:1/18:2/18:2) |
| CL(70:7) | [M-2H]^2-^ | 710.4710 | 710.4711 | 0.2 | 2.6 | (16:1/18:2/18:2/18:2)  (16:2/18:1/18:2/18:2) |
| CL(72:5) | [M-2H]^2-^ | 726.5023 | 726.4988 | -4.9 | 2.7 | (18:0/18:1/18:2/18:2) |
| CL(72:6) | [M-2H]^2-^ | 725.4945 | 725.4915 | -4.2 | 2.6 | (18:0/18:1/18:2/18:3) |
| CL(72:7) | [M-2H]^2-^ | 724.4840 | 724.4828 | -1.6 | 2.6 | (18:0/18:2/18:2/18:3)  (18:1/18:2/18:2/18:2) |
| CL(72:8) | [M-2H]^2-^ | 723.4788 | 723.4796 | 1.1 | 2.6 | (18:2/18:2/18:2/18:2) |
| CL(72:9) | [M-2H]^2-^ | 722.4710 | 722.4731 | 2.9 | 2.6 | (18:2/18:2/18:2/18:3) |
| CL(74:10) | [M-2H]^2-^ | 735.4788 | 735.4805 | 2.4 | 2.6 | (18:2/18:2/18:2/20:4) |
| CL(74:11) | [M-2H]^2-^ | 734.4710 | 734.4729 | 2.5 | 2.6 | (16:1/18:2/18:2/22:6) |
| CL(74:8) | [M-2H]^2-^ | 737.4945 | 737.4931 | -1.8 | 2.6 | (18:1/18:2/18:3/20:2)  (18:1/18:1/18:3/20:3) |
| CL(74:9) | [M-2H]^2-^ | 736.4867 | 736.4852 | -2.1 | 2.6 | (18:2/18:2/18:3/20:2) |
| CL(76:10) | [M-2H]^2-^ | 749.4945 | 749.4915 | -3.9 | 2.6 | (16:0/16:0/22:5/22:5)  (18:1/18:1/18:2/22:6)  (18:0/18:2/18:2/22:6) |
| CL(76:11) | [M-2H]^2-^ | 748.4867 | 748.4856 | -1.5 | 2.6 | (16:0/16:0/22:5/22:6)  (16:0/18:0/20:5/22:6)  (18:1/18:2/18:2/22:6) |
| CL(76:12) | [M-2H]^2-^ | 747.4788 | 747.4807 | 2.6 | 2.6 | (16:0/16:0/22:6/22:6)  (18:1/18:2/18:3/22:6)  (18:0/18:3/18:3/22:6) |
| CL(76:13) | [M-2H]^2-^ | 746.4710 | 746.4702 | -1.1 | 2.6 | (16:0/16:1/22:6/22:6)  (18:0/18:3/20:5/20:5)  (18:1/18:2/20:5/20:5) |
| LPC(16:0) | [M+H]^+^ | 496.3398 | 496.3411 | 2.6 | 12.6 | - |
| LPC(16:1) | [M+H]^+^ | 494.3241 | 494.3253 | 2.4 | 12.7 | (16:1) |
| LPC(18:0) | [M+H]^+^ | 524.3711 | 524.3724 | 2.4 | 12.1 | (18:0) |
| LPC(18:1) | [M+H]^+^ | 522.3554 | 522.3574 | 3.8 | 12.3 | (18:1) |
| LPC(18:2) | [M+H]^+^ | 520.3398 | 520.3375 | -4.3 | 12.4 | (18:2) |
| LPC(18:3) | [M+H]^+^ | 518.3241 | 518.3232 | -1.8 | 12.6 | (18:3) |
| LPC(20:3) | [M+H]^+^ | 546.3554 | 546.3548 | -1.0 | 12.1 | - |
| LPC(20:4) | [M+H]^+^ | 544.3398 | 544.3400 | 0.4 | 11.3 | (20:4) |
| LPC(20:5) | [M+H]^+^ | 542.3241 | 542.3235 | -1.2 | 12.4 | (20:5) |
| LPC(22:4) | [M+H]^+^ | 572.3711 | 572.3705 | -1.1 | 11.8 | - |
| LPC(22:5) | [M+H]^+^ | 570.3554 | 570.3554 | 0.1 | 11.3 | - |
| LPC(22:6) | [M+H]^+^ | 568.3398 | 568.3415 | 3.0 | 11.2 | (22:6) |
| LPCp(16:1) | [M+H]^+^ | 480.3448 | 480.3460 | 2.5 | 12.0 | - |
| PA(36:2) | [M-H]- | 699.4952 | 699.497 | 0.0 | 3.8 | (16:0/20:2)  (18:0/18:2)  (18:1/18:1) |
| PC(32:0) | [M+H]^+^ | 734.5694 | 734.5704 | 1.4 | 8.8 | (16:0/16:0) |
| PC(32:1) | [M+H]^+^ | 732.5538 | 732.5517 | -2.8 | 8.8 | (16:0/16:1) |
| PC(32:2) | [M+H]^+^ | 730.5381 | 730.5401 | 2.7 | 8.7 | (16:1/16:1) |
| PC(34:1) | [M+H]^+^ | 760.5851 | 760.5838 | -1.8 | 8.6 | (16:0/18:1)  (16:1/18:0) |
| PC(34:2) | [M+H]^+^ | 758.5694 | 758.5715 | 2.7 | 8.6 | (16:0/18:2) |
| PC(34:3) | [M+H]^+^ | 756.5538 | 756.5558 | 2.7 | 8.6 | (16:1/18:2)  (16:0/18:3) |
| PC(34:4) | [M+H]^+^ | 754.5381 | 754.5393 | 1.5 | 8.5 | - |
| PC(34:5) | [M+H]^+^ | 752.5225 | 752.5229 | 0.6 | 8.6 | - |
| PC(36:1) | [M+H]^+^ | 788.6164 | 788.6143 | -2.7 | 8.4 | (18:1/18:0) |
| PC(36:2) | [M+H]^+^ | 786.6007 | 786.6019 | 1.5 | 8.4 | (18:0/18:2)  (18:1/18:1) |
| PC(36:3) | [M+H]^+^ | 784.5851 | 784.5852 | 0.2 | 8.4 | (16:1/20:2)  (18:1/18:2) |
| PC(36:4) | [M+H]^+^ | 782.5694 | 782.5713 | 2.4 | 8.2 | (16:0/20:4)  (18:2/18:2) |
| PC(36:5) | [M+H]^+^ | 780.5538 | 780.5543 | 0.6 | 8.3 | (16:0/20:5) |
| PC(36:6) | [M+H]^+^ | 778.5381 | 778.5406 | 3.2 | 8.3 | - |
| PC(38:2) | [M+H]^+^ | 814.6320 | 814.6316 | -0.6 | 8.3 | (16:1/22:1)  (18:0/20:2)  (18:1/20:1)  (18:2/20:0) |
| PC(38:4) | [M+H]^+^ | 810.6007 | 810.6038 | 3.9 | 8.0 | (16:0/22:4)  (18:0/20:4) |
| PC(38:5) | [M+H]^+^ | 808.5851 | 808.5842 | -1.1 | 8.1 | (16:0/22:5)  (18:0/20:5)  (18:1/20:4) |
| PC(38:6) | [M+H]^+^ | 806.5694 | 806.5717 | 2.9 | 8.1 | (16:0/22:6)  (16:1/22:5)  (18:1/20:5) |
| PC(38:7) | [M+H]^+^ | 804.5538 | 804.5541 | 0.4 | 8.1 | (16:1/22:6) |
| PC(38:8) | [M+H]^+^ | 802.5381 | 802.5384 | 0.3 | 8.2 | (16:2/22:6)  (20:4/18:4) |
| PC(40:4) | [M+H]^+^ | 838.6320 | 838.6327 | 0.9 | 8.1 | (16:0/24:4)  (18:0/22:4)  (20:0/20:4) |
| PC(40:5) | [M+H]^+^ | 836.6164 | 836.6158 | -0.7 | 8.0 | (16:0/24:5)  (18:0/22:5)  (20:2/20:3)  (20:1/20:4) |
| PC(40:6) | [M+H]^+^ | 834.6007 | 834.6028 | 2.5 | 7.9 | (18:0/22:6) (16:0/24:6)  (18:1/22:5) |
| PC(40:7) | [M+H]^+^ | 832.5851 | 832.5862 | 1.3 | 7.9 | (18:1/22:6)  (18:2/22:5) |
| PC(40:8) | [M+H]^+^ | 830.5694 | 830.5705 | 1.3 | 8.0 | (18:2/22:6) |
| PC(40:9) | [M+H]^+^ | 828.5538 | 828.5524 | -1.6 | 8.0 | (18:3/22:6)  (20:4/20:5) |
| PC(42:10) | [M+H]^+^ | 854.5694 | 854.5670 | -2.8 | 7.8 | - |
| PC(42:11) | [M+H]^+^ | 852.5538 | 852.5523 | -1.7 | 7.9 | - |
| PC(42:6) | [M+H]^+^ | 862.6320 | 862.6308 | -1.3 | 7.9 | - |
| PC(42:7) | [M+H]^+^ | 860.6164 | 860.6170 | 0.7 | 7.9 | (16:1/26:6) |
| PC(42:8) | [M+H]^+^ | 858.6007 | 858.5991 | -1.9 | 7.9 | - |
| PC(42:9) | [M+H]^+^ | 856.5851 | 856.5833 | -2.2 | 7.8 | - |
| PC(44:10) | [M+H]^+^ | 882.6007 | 882.5990 | -2.0 | 7.7 | - |
| PC(44:11) | [M+H]^+^ | 880.5851 | 880.5857 | 0.7 | 7.7 | - |
| PC(44:12) | [M+H]^+^ | 878.5694 | 878.5733 | 4.4 | 7.6 | - |
| PCp(32:0) | [M+H]^+^ | 720.5902 | 720.5904 | 0.3 | 9.0 | (p16:0/16:0) |
| PCp(32:1) | [M+H]^+^ | 718.5745 | 718.5760 | 2.1 | 8.7 | (p16:1/16:0) |
| PCp(34:0) | [M+H]^+^ | 748.6215 | 748.6198 | -2.2 | 8.8 | (p18:1/16:0) |
| PCp(34:1) | [M+H]^+^ | 746.6058 | 746.6067 | 1.2 | 8.8 | (p18:1/16:0/)  (p16:0/18:1) |
| PCp(34:2) | [M+H]^+^ | 744.5902 | 744.5918 | 2.2 | 8.6 | (p16:0/18:2) |
| PCp(34:3) | [M+H]^+^ | 742.5745 | 742.5755 | 1.4 | 8.5 | (p16:1/18:2) |
| PCp(36:2) | [M+H]^+^ | 772.6215 | 772.6213 | -0.3 | 8.5 | (p18:0/18:2) |
| PCp(36:3) | [M+H]^+^ | 770.6058 | 770.6062 | 0.5 | 8.4 | (p18:1/18:2) |
| PCp(36:4) | [M+H]^+^ | 768.5902 | 768.5894 | -1.0 | 8.4 | (p16:0/20:4) |
| PCp(36:5) | [M+H]^+^ | 766.5745 | 766.5761 | 2.1 | 8.1 | - |
| PCp(36:6) | [M+H]^+^ | 764.5589 | 764.5577 | -1.6 | 8.2 | - |
| PCp(38:5) | [M+H]^+^ | 794.6058 | 794.6040 | -2.3 | 8.2 | (p18:1/20:4)  (p16:0/22:5) |
| PCp(38:6) | [M+H]^+^ | 792.5902 | 792.5911 | 1.2 | 8.1 | (p16:0/22:6) |
| PCp(38:7) | [M+H]^+^ | 790.5745 | 790.5767 | 2.8 | 8.0 | - |
| PCp(40:6) | [M+H]^+^ | 820.6215 | 820.6220 | 0.7 | 8.0 | (p18:0/22:6) |
| PCp(40:7) | [M+H]^+^ | 818.6058 | 818.6034 | -2.9 | 7.9 | (p18:1/22:6) |
| PE(32:0) | [M+H]^+^ | 692.5225 | 692.5229 | 0.6 | 4.5 | - |
| PE(32:1) | [M+H]^+^ | 690.5068 | 690.5070 | 0.3 | 4.4 | (16:1/16:0) |
| PE(34:1) | [M+H]^+^ | 718.5381 | 718.5359 | -3.1 | 4.4 | (16:0/18:1)  (16:1/18:0) |
| PE(34:2) | [M+H]^+^ | 716.5225 | 716.5241 | 2.2 | 4.4 | (16:0/18:2) |
| PE(34:3) | [M+H]^+^ | 714.5068 | 714.5090 | 3.1 | 4.4 | (16:1/18:2) |
| PE(36:2) | [M+H]^+^ | 744.5538 | 744.5565 | 3.6 | 4.4 | (18:0/18:2)  (18:1/18:1) |
| PE(36:3) | [M+H]^+^ | 742.5400 | 742.5396 | -0.6 | 4.4 | (18:1/18:2) |
| PE(36:4) | [M+H]^+^ | 740.5225 | 740.5243 | 2.4 | 4.3 | - |
| PE(36:5) | [M+H]^+^ | 738.5068 | 738.5079 | 1.5 | 4.4 | (16:0/20:5) |
| PE(36:6) | [M+H]^+^ | 736.4912 | 736.4920 | 1.1 | 4.4 | - |
| PE(38:4) | [M+H]^+^ | 768.5538 | 768.5545 | 0.9 | 4.2 | (18:0/20:4) |
| PE(38:5) | [M+H]^+^ | 766.5381 | 766.5364 | -2.3 | 4.3 | (16:0/22:5)  (18:1/20:4)  (18:0/20:5) |
| PE(38:6) | [M+H]^+^ | 764.5225 | 764.5242 | 2.2 | 4.2 | (16:0/22:6)  (18:2/20:4) |
| PE(38:7) | [M+H]^+^ | 762.5068 | 762.5086 | 2.3 | 4.3 | (16:1/22:6) |
| PE(38:8) | [M+H]^+^ | 760.4912 | 760.4935 | 3.0 | 4.4 | (18:4/20:4) |
| PE(40:10) | [M+H]^+^ | 784.4912 | 784.4916 | 0.5 | 4.6 | - |
| PE(40:4) | [M+H]^+^ | 796.5851 | 796.5860 | 1.1 | 4.2 | (18:0/22:4) |
| PE(40:5) | [M+H]^+^ | 794.5694 | 794.5660 | -4.3 | 4.2 | - |
| PE(40:6) | [M+H]^+^ | 792.5538 | 792.5567 | 3.7 | 4.2 | (18:0/22:6) |
| PE(40:7) | [M+H]^+^ | 790.5381 | 790.5390 | 1.1 | 4.3 | (18:1/22:6)  (18:2/22:5) |
| PE(40:8) | [M+H]^+^ | 788.5225 | 788.5212 | -1.6 | 4.2 | (18:2/22:6) |
| PE(40:9) | [M+H]^+^ | 786.5068 | 786.5047 | -2.7 | 4.3 | - |
| PE(42:10) | [M+H]^+^ | 812.5225 | 812.5213 | -1.4 | 4.2 | - |
| PE(42:11) | [M+H]^+^ | 810.5068 | 810.5038 | -3.7 | 4.3 | - |
| PE(42:7) | [M+H]^+^ | 818.5694 | 818.5704 | 1.2 | 4.2 | - |
| PE(42:8) | [M+H]^+^ | 816.5538 | 816.5522 | -2.0 | 4.2 | - |
| PE(42:9) | [M+H]^+^ | 814.5381 | 814.5364 | -2.1 | 4.2 | - |
| PEp(34:1) | [M+H]^+^ | 704.5589 | 704.5589 | -0.1 | 4.4 | (p18:1/16:0)  (p16:1/18:0) |
| PEp(34:2) | [M+H]^+^ | 702.5432 | 702.5403 | -4.2 | 4.4 | (p18:1/16:1)  (p16:1/18:1) |
| PEp(34:3) | [M+H]^+^ | 700.5276 | 700.5289 | 1.9 | 4.4 | (p16:1/18:2) |
| PEp(36:2) | [M+H]^+^ | 730.5745 | 730.5748 | 0.4 | 4.4 | - |
| PEp(36:3) | [M+H]^+^ | 728.5589 | 728.5604 | 2.1 | 4.4 | (p18:1/18:2) |
| PEp(36:4) | [M+H]^+^ | 726.5432 | 726.5437 | 0.7 | 4.4 | - |
| PEp(36:5) | [M+H]^+^ | 724.5276 | 724.5298 | 3.0 | 4.3 | (p16:1/20:4) |
| PEp(38:3) | [M+H]^+^ | 756.5902 | 756.5905 | 0.4 | 4.4 | - |
| PEp(38:4) | [M+H]^+^ | 754.5745 | 754.5718 | -3.6 | 4.3 | (p18:0/20:4) |
| PEp(38:5) | [M+H]^+^ | 752.5589 | 752.5592 | 0.4 | 4.3 | (p18:1/20:4) |
| PEp(38:6) | [M+H]^+^ | 750.5432 | 750.5403 | -3.8 | 4.3 | (p16:1/22:5)  (p16:0/22:6) |
| PEp(38:7) | [M+H]^+^ | 748.5276 | 748.5297 | 2.8 | 4.2 | (p16:1/22:6) |
| PEp(38:8) | [M+H]^+^ | 746.5125 | 746.5112 | -1.7 | 4.3 | - |
| PEp(40:10) | [M+H]^+^ | 770.5125 | 770.5110 | -1.9 | 4.2 | - |
| PEp(40:5) | [M+H]^+^ | 780.5902 | 780.5872 | -3.8 | 4.2 | - |
| PEp(40:6) | [M+H]^+^ | 778.5745 | 778.5778 | 4.3 | 4.2 | - |
| PEp(40:7) | [M+H]^+^ | 776.5589 | 776.5607 | 2.4 | 4.2 | (p18:1/22:6) |
| PEp(40:8) | [M+H]^+^ | 774.5438 | 774.5447 | 1.2 | 4.2 | - |
| PEp(40:9) | [M+H]^+^ | 772.5281 | 772.5266 | -2.0 | 4.2 | - |
| PEp(42:10) | [M+H]^+^ | 798.5438 | 798.5412 | -3.2 | 4.2 | - |
| LPE(16:0) | [M+H]^+^ | 454.2940 | 454.2942 | 0.3 | 5.3 | (16:0) |
| LPE(16:1) | [M+H]^+^ | 452.2783 | 452.2783 | 0.0 | 5.3 | (16:1) |
| LPE(18:0) | [M+H]^+^ | 482.3253 | 482.3257 | 0.8 | 5.2 | (18:0) |
| LPE(18:1) | [M+H]^+^ | 480.3098 | 480.3098 | 0.0 | 5.2 | (18:1) |
| LPE(18:2) | [M+H]^+^ | 478.2940 | 478.2940 | 0.0 | 5.2 | (18:2) |
| LPE(20:3) | [M+H]^+^ | 504.3072 | 504.3072 | 0.0 | 5.2 | - |
| LPE(20:4) | [M+H]^+^ | 502.2938 | 502.2938 | 0.0 | 5.1 | (20:4) |
| LPE(20:5) | [M+H]^+^ | 500.2768 | 500.2769 | 0.2 | 5.2 | - |
| LPE(22:4) | [M+H]^+^ | 530.3222 | 530.3222 | 0.1 | 5.0 | - |
| LPE(22:5) | [M+H]^+^ | 528.3083 | 528.3083 | 0.0 | 5.0 | - |
| LPE(22:6) | [M+H]^+^ | 526.2943 | 526.2943 | 0.0 | 5.0 | (22:6) |
| LPEp(16:0) | [M+H]^+^ | 438.2988 | 438.2988 | 0.0 | 5.2 | - |
| LPEp(18:0) | [M+H]^+^ | 466.3305 | 466.3305 | 0.0 | 5.1 | - |
| PG(32:0) | [M-H]- | 721.5025 | 721.5038 | 1.8 | 2.8 | (16:0/16:0) |
| PG(34:0) | [M-H]- | 749.5338 | 749.5303 | -4.7 | 2.8 | (16:0/18:0) |
| PG(34:1) | [M-H]- | 747.5182 | 747.5202 | 2.7 | 2.8 | (16:0/18:1) |
| PG(34:2) | [M-H]- | 745.5025 | 745.5041 | 2.2 | 2.8 | (16:0/18:2)  (16:1/18:1) |
| PG(36:0) | [M-H]- | 777.5651 | 777.5637 | -1.8 | 2.8 | - |
| PG(36:1) | [M-H]- | 775.5495 | 775.5495 | 0.0 | 2.8 | (18:0/18:1) |
| PG(36:2) | [M-H]- | 773.5338 | 773.5335 | -0.4 | 2.8 | (18:0/18:2) |
| PG(36:3) | [M-H]- | 771.5182 | 771.5188 | 0.8 | 2.8 | (18:1/18:2) |
| PG(36:4) | [M-H]- | 769.5025 | 769.5033 | 1.0 | 2.8 | (18:2/18:2) |
| PG(38:4) | [M-H]- | 797.5338 | 797.5345 | 0.9 | 2.8 | (18:1/20:3) |
| PG(38:5) | [M-H]- | 795.5182 | 795.5184 | 0.2 | 2.8 | (18:2/20:3)  (18:3/20:2) |
| PG(38:6) | [M-H]- | 793.5025 | 793.5053 | 3.6 | 2.8 | (18:2/20:4) |
| PG(40:6) | [M-H]- | 821.5338 | 821.5355 | 2.1 | 2.8 | (18:0/22:6) |
| PG(40:7) | [M-H]- | 819.5182 | 819.5188 | 0.7 | 2.7 | (18:1/22:6)  (18:2:22:5) |
| PG(40:8) | [M-H]- | 817.5025 | 817.5040 | 1.9 | 2.7 | (18:2/22:6) |
| PI(34:1) | [M-H]- | 835.5342 | 835.5361 | 2.3 | 2.8 | (16:0/18:1)  (16:1/18:0) |
| PI(34:2) | [M-H]- | 833.5186 | 833.5204 | 2.1 | 2.8 | (16:0/18:2)  (16:1/18:1) |
| PI(36:1) | [M-H]- | 863.5655 | 863.5668 | 1.5 | 2.8 | (18:0/18:1) |
| PI(36:2) | [M-H]- | 861.5499 | 861.5510 | 1.3 | 2.8 | (18:0/18:2)  (18:1/18:1) |
| PI(36:3) | [M-H]- | 859.5342 | 859.5309 | -3.8 | 2.8 | (16:0/20:3)  (16:1/20:2)  (18:0/18:3)  (18:1/18:2) |
| PI(36:4) | [M-H]- | 857.5186 | 857.5207 | 2.4 | 2.8 | (16:0/20:4) |
| PI(38:3) | [M-H]- | 887.5655 | 887.5659 | 0.4 | 2.8 | (16:0/22:3) |
| PI(38:4) | [M-H]- | 885.5499 | 885.5523 | 2.8 | 2.8 | (18:0/20:4)  (18:2/20:2) |
| PI(38:5) | [M-H]- | 883.5342 | 883.5359 | 1.9 | 2.8 | (16:0/20:5)  (18:1/20:4)  (18:2/20:3) |
| PI(38:6) | [M-H]- | 881.5186 | 881.5209 | 2.7 | 2.8 | (18:2/20:6) |
| PI(40:4) | [M-H]- | 913.5812 | 913.5788 | -2.6 | 2.8 | - |
| PI(40:5) | [M-H]- | 911.5655 | 911.5651 | -0.4 | 2.8 | (16:0/22:5) |
| PI(40:6) | [M-H]- | 909.5499 | 909.5521 | 2.4 | 2.8 | (18:0/22:6) |
| PI(40:7) | [M-H]- | 907.5342 | 907.5363 | 2.3 | 2.8 | (18:1/22:6) |
| LPI(16:0) | [M-H]- | 571.2889 | 571.2905 | 2.8 | 2.9 | (16:0) |
| LPI(18:0) | [M-H]- | 599.3202 | 599.3222 | 3.3 | 2.9 | (18:0) |
| PS(34:1) | [M-H]- | 760.5134 | 760.5159 | 3.3 | 5.1 | (16:0/18:1)  (16:1/18:0) |
| PS(34:2) | [M-H]- | 758.4978 | 758.4982 | 0.5 | 5.1 | (16:0/18:2)  (16:1/18:1)  (16:2/18:0) |
| PS(36:1) | [M-H]- | 788.5447 | 788.5470 | 2.9 | 5.3 | (18:0/18:1) |
| PS(36:2) | [M-H]- | 786.5291 | 786.5327 | 4.6 | 5.3 | (18:0/18:2)  (18:1/18:1) |
| PS(36:3) | [M-H]- | 784.5134 | 784.5169 | 4.4 | 5.1 | - |
| PS(36:4) | [M-H]- | 782.4978 | 782.5003 | 3.3 | 5.0 | - |
| PS(38:2) | [M-H]- | 814.5604 | 814.5592 | -1.5 | 5.1 | (16:0/22:2)  (16:1/22:1)  (18:2/20:0) |
| PS(38:3) | [M-H]- | 812.5447 | 812.5453 | 0.7 | 5.0 | (16:1/22:2)  (18:2/20:1) |
| PS(38:4) | [M-H]- | 810.5291 | 810.5303 | 1.5 | 4.9 | (16:0/22:4)  (18:0/20:4)  (18:2/20:2) |
| PS(38:5) | [M-H]- | 808.5134 | 808.5134 | 0.0 | 5.0 | - |
| PS(38:6) | [M-H]- | 806.4978 | 806.5003 | 3.1 | 4.9 | (16:0/22:6)  (18:2/20:4)  (18:4/20:2) |
| PS(40:4) | [M-H]- | 838.5604 | 838.5608 | 0.5 | 5.0 | (20:0/20:4)  (16:1/24:3)  (18:0/22:4)  (18:2/22:2)  (20:1/20:3)/ |
| PS(40:5) | [M-H]- | 836.5447 | 836.5439 | -0.9 | 4.9 | (18:0/22:5)  (16:0/24:5)  (18:2/22:3) |
| PS(40:6) | [M-H]- | 834.5291 | 834.5308 | 2.1 | 4.9 | (16:0/24:6)  (18:0/22:6)  (18:2/22:4)  (20:1/20:5)  (20:2/20:4) |
| PS(40:7) | [M-H]- | 832.5134 | 832.5151 | 2.1 | 4.9 | (18:1/22:6)  (18:2/22:5)  (18:3/22:4) |
| PS(42:7) | [M-H]- | 860.5447 | 860.5440 | -0.9 | 4.9 | (18:1/24:6)  (18:2/24:5)  (18:3/24:4)  (20:1/22:6) |
| PS(42:8) | [M-H]- | 858.5291 | 858.5281 | -1.2 | 4.4 | (18:2/24:6)  (20:2/22:6)  (20:3/22:5) |
| PS(42:9) | [M-H]- | 856.5134 | 856.5127 | -0.8 | 5.0 | (20:3/22:6)  (20:4/22:5)  (20:5/22:4) |
| SM(d34:1) | [M+H]^+^ | 703.5748 | 703.5762 | 1.9 | 10.4 | (d18:1/16:0) |
| SM(d36:1) | [M+H]^+^ | 731.6061 | 731.6079 | 2.5 | 10.2 | (d18:1/18:0) |
| SM(d36:2) | [M+H]^+^ | 729.5905 | 729.5923 | 2.5 | 10.2 | (d18:1/18:1) |
| SM(d38:1) | [M+H]^+^ | 759.6374 | 759.6386 | 1.5 | 10.1 | (d18:1/20:0) |
| SM(d38:2) | [M+H]^+^ | 757.6218 | 757.6234 | 2.1 | 10.0 | (d18:1/20:1) |
| SM(d40:0) | [M+H]^+^ | 789.6844 | 789.6812 | -4.0 | 9.7 | - |
| SM(d40:1) | [M+H]^+^ | 787.6687 | 787.6688 | 0.1 | 9.8 | (d18:1/22:0) |
| SM(d40:2) | [M+H]^+^ | 785.6531 | 785.6537 | 0.7 | 9.9 | - |
| SM(d42:1) | [M+H]^+^ | 815.7000 | 815.7015 | 1.8 | 9.6 | - |
| SM(d42:2) | [M+H]^+^ | 813.6844 | 813.6863 | 2.4 | 9.7 | - |
| SM(d42:3) | [M+H]^+^ | 811.6687 | 811.6688 | 0.1 | 9.7 | - |

:

Supplementary Table S 2: Results of the Kluskal-Wallis univariate analysis. All the PL species are detailed and ranked by its p value. Species with p value under 0.05 are marked with shading.

| PL specie | p value |
| --- | --- |
| PS(34:1) | 0.003 | SM(d38:1) | 0.081 | PC(38:6) | 0.387 |
| CL(74:11 ) | 0.003 | PS(38:5) | 0.082 | PS(42:8) | 0.390 |
| CL(70:7 ) | 0.004 | PS(38:6) | 0.083 | SM(d34:1) | 0.390 |
| CL(76:13 ) | 0.004 | SM(d42:2) | 0.083 | PC(38:5) | 0.392 |
| CL(72:9 ) | 0.004 | PE(p36:3) | 0.085 | Cer(d36:2)) | 0.400 |
| CL(74:9 ) | 0.004 | LPE(18:2) | 0.085 | PE(p36:5) | 0.402 |
| CL(74:10 ) | 0.004 | PC(42:8) | 0.088 | PE(34:2) | 0.408 |
| CL(70:6 ) | 0.004 | LPE(20:5) | 0.089 | Cer(d40:1) | 0.412 |
| CL(76:11 ) | 0.005 | LPE(p18:0) | 0.089 | PE(36:3) | 0.414 |
| CL(76:12 ) | 0.005 | PC(44:11) | 0.091 | LPC(16:0) | 0.416 |
| CL(74:8 ) | 0.005 | PC(p32:1) | 0.093 | Cer(d34:1) | 0.419 |
| CL(72:5 ) | 0.005 | PC(p36:2) | 0.095 | LPC(18:0) | 0.428 |
| CL(72:6 ) | 0.005 | PC(p36:6) | 0.095 | PC(p34:3) | 0.428 |
| CL(72:7 ) | 0.005 | PC(42:6) | 0.107 | LPC(18:3) | 0.436 |
| CL(72:8 ) | 0.005 | LPE(18:0) | 0.107 | PE(42:7) | 0.440 |
| PS(36:1) | 0.005 | PI(34:2) | 0.112 | PE(p34:1) | 0.455 |
| CL(70:5 ) | 0.005 | PC(p36:3) | 0.114 | PC(40:8) | 0.472 |
| CL(76:10 ) | 0.005 | PG(36:2) | 0.114 | LPC(16:1) | 0.480 |
| CL(68:6 ) | 0.006 | PC(38:2) | 0.117 | PC(34:3) | 0.480 |
| PS(38:2) | 0.007 | PC(36:4) | 0.119 | PE(p40:10) | 0.494 |
| PS(36:3) | 0.008 | LPE(18:1) | 0.128 | PE(40:7) | 0.498 |
| PS(38:3) | 0.010 | PS(36:2) | 0.132 | LPC(20:3) | 0.508 |
| CL(68:5 ) | 0.015 | PG(38:4) | 0.132 | PC(34:1) | 0.516 |
| LPI(16:0) | 0.016 | SM(d40:1) | 0.136 | PC(p38:6) | 0.519 |
| PC(42:10) | 0.021 | PE(p42:10) | 0.138 | LPC(p16:1) | 0.533 |
| PS(36:4) | 0.023 | PE(p40:6) | 0.138 | PE(40:5) | 0.538 |
| PC(p32:0) | 0.024 | PG(36:4) | 0.140 | PE(40:10) | 0.569 |
| LPI(18:0) | 0.027 | PI(36:3) | 0.142 | PE(40:4) | 0.578 |
| PI(40:5) | 0.029 | LPC(22:5) | 0.144 | PE(p38:4) | 0.588 |
| PI(36:1) | 0.029 | PG(34:0) | 0.147 | PE(38:5) | 0.594 |
| PC(42:11) | 0.029 | PC(38:4) | 0.148 | PC(40:6) | 0.596 |
| PI(38:4) | 0.032 | LPE(p16:0) | 0.159 | PE(40:8) | 0.596 |
| PC(p36:5) | 0.033 | SM(d40:0) | 0.163 | PC(32:2) | 0.605 |
| PI(38:5) | 0.035 | LPE(20:4) | 0.165 | PE(p34:3) | 0.617 |
| PC(p34:0) | 0.036 | LPC(22:6) | 0.165 | LPC(20:5) | 0.628 |
| PC(42:9) | 0.037 | PG(36:1) | 0.168 | PC(38:8) | 0.629 |
| PI(34:1) | 0.037 | PE(38:7) | 0.168 | Cer(d38:2) | 0.644 |
| PC(44:10) | 0.037 | LPE(20:3) | 0.169 | PC(32:1) | 0.651 |
| PG(40:6) | 0.037 | PC(40:7) | 0.195 | PE(38:6) | 0.656 |
| PI(38:3) | 0.038 | PC(p40:6) | 0.202 | PE(38:4) | 0.657 |
| PC(32:0) | 0.039 | LPC(20:4) | 0.205 | PE(32:0) | 0.664 |
| PI(40:4) | 0.040 | PE(34:3) | 0.215 | PC(34:4) | 0.670 |
| PI(40:6) | 0.046 | PG(40:8) | 0.227 | PE(p40:5) | 0.683 |
| PS(42:9) | 0.047 | LPC(18:1) | 0.227 | PS(40:4) | 0.703 |
| PS(40:6) | 0.047 | PG(38:5) | 0.231 | PE(p40:9) | 0.706 |
| SM(d38:2) | 0.051 | PG(36:3) | 0.234 | PE(36:5) | 0.717 |
| PS(42:7) | 0.055 | PC(38:7) | 0.243 | PE(40:9) | 0.739 |
| PI(36:4) | 0.056 | PE(p38:5) | 0.243 | PC(34:2) | 0.744 |
| SM(d36:2) | 0.057 | LPC(22:4) | 0.247 | Cer(d38:1) | 0.758 |
| PE(p36:2) | 0.058 | PS(34:2) | 0.250 | PC(34:5) | 0.767 |
| PS(40:5) | 0.059 | PC(40:4) | 0.250 | PS(40:7) | 0.774 |
| PA(36:2) | 0.062 | PG(36:0) | 0.262 | PE(42:8) | 0.780 |
| LPE(16:1) | 0.063 | Cer(d36:1) | 0.263 | PE(p34:2) | 0.783 |
| PE(36:6) | 0.063 | PC(44:12) | 0.265 | PE(36:2) | 0.783 |
| LPE(16:0) | 0.064 | PG(34:1) | 0.265 | PE(40:6) | 0.797 |
| LPE(22:6) | 0.064 | PG(32:0) | 0.271 | PE(32:1) | 0.798 |
| SM(d40:2) | 0.065 | PC(p40:7) | 0.271 | PE(p40:7) | 0.841 |
| PC(40:9) | 0.065 | Cer(d42:2) | 0.277 | PC(p38:7) | 0.866 |
| PI(38:6) | 0.065 | PC(36:1) | 0.278 | PE(p40:8) | 0.877 |
| PE(p38:3) | 0.066 | PE(p36:4) | 0.280 | PE(36:4) | 0.881 |
| PI(40:7) | 0.066 | PC(36:3) | 0.284 | PE(42:9) | 0.919 |
| PI(36:2) | 0.070 | PC(40:5) | 0.294 | PE(42:11) | 0.940 |
| PC(p34:1) | 0.071 | PG(38:6) | 0.304 | PC(36:6) | 0.944 |
| PC(p34:2) | 0.072 | PC(36:2) | 0.305 | PE(p38:7) | 0.951 |
| PS(38:4) | 0.073 | PE(p38:8) | 0.321 | PE(p38:6) | 0.955 |
| SM(d42:1) | 0.073 | PC(p36:4) | 0.326 | PE(34:1) | 0.957 |
| PC(42:7) | 0.076 | SM(d36:1) | 0.350 | PC(36:5) | 0.962 |
| PC(p38:5) | 0.076 | LPC(18:2) | 0.353 | PE(42:10) | 0.968 |
| LPE(22:5) | 0.078 | Cer(d42:1) | 0.369 | PG(40:7) | 0.998 |
| SM(d42:3) | 0.078 | PE(38:8) | 0.377 |  |  |
| LPE(22:4) | 0.079 | PG(34:2) | 0.381 |  |  |

Supplementary Table S 3: Species showing significant differences (p value<0.05) and up- or downregulation in the pairwise phospholipid expression profiling analysis (Wilcoxon Mann-Whitney Pairwise test, Figure 4 of the maisn manuscript). Comparison between CTex and Ctsed, BBNsed and CTsed, and BBN and BBNex are included in the table, together with the sense of the variation (up/down).

| BBNex vs CTsed | | | BBNsed vs Ctsed | | | BBNex vs BBNsed | |
| --- | --- | --- | --- | --- | --- | --- | --- |
| PL specie | Regulation | PL specie | | Regulation | PL specie | | Regulation |
| CL(70:5) | DOWN | PS(34:1) | | UP | PA(36:2) | | DOWN |
|  |  | PS(36:1) | | UP | PS(34:1) | | DOWN |
|  |  | PS(38:3) | | UP | PS(36:1) | | DOWN |
|  |  | PS(38:2) | | UP | PS(36:3) | | DOWN |
|  |  | PS(36:3) | | UP | PS(36:4) | | DOWN |
|  |  | PS(42:9) | | DOWN | PS(38:2) | | DOWN |
|  |  | PS(40:5) | | DOWN | PS(38:3) | | DOWN |
|  |  | PS(40:6) | | DOWN | CL(68:6 ) | | UP |
|  |  | PI(40:6) | | DOWN | CL(70:5 ) | | UP |
|  |  | PI(38:5) | | DOWN | CL(70:6 ) | | UP |
|  |  | PI(38:6) | | DOWN | CL(70:7 ) | | UP |
|  |  | PI(38:4) | | DOWN | CL(72:5 ) | | UP |
|  |  | PI(40:7) | | DOWN | CL(72:6 ) | | UP |
|  |  | PS(42:7) | | DOWN | CL(72:7 ) | | UP |
|  |  | PI(34:1) | | DOWN | CL(72:8 ) | | UP |
|  |  | PI(36:1) | | DOWN | CL(72:9 ) | | UP |
|  |  | LPI(18:0) | | DOWN | CL(74:10 ) | | UP |
|  |  | CL(70:5 ) | | DOWN | CL(74:11 ) | | UP |
|  |  | CL(68:5 ) | | DOWN | CL(74:8 ) | | UP |
|  |  | CL(72:5 ) | | DOWN | CL(74:9 ) | | UP |
|  |  | CL(70:6 ) | | DOWN | CL(76:10 ) | | UP |
|  |  | CL(74:10 ) | | DOWN | CL(76:11 ) | | UP |
|  |  | CL(74:9 ) | | DOWN | CL(76:12 ) | | UP |
|  |  | CL(76:12 ) | | DOWN | CL(76:13 ) | | UP |
|  |  | CL(70:7 ) | | DOWN | LPC(22:5) | | UP |
|  |  | CL(72:6 ) | | DOWN | LPE(16:0) | | UP |
|  |  | CL(76:11 ) | | DOWN | LPE(16:1) | | UP |
|  |  | CL(76:10 ) | | DOWN | LPE(18:0) | | UP |
|  |  | CL(68:6 ) | | DOWN | LPE(18:1) | | UP |
|  |  | CL(72:7 ) | | DOWN | LPE(18:2) | | UP |
|  |  | CL(72:8 ) | | DOWN | LPE(20:4) | | UP |
|  |  | CL(74:11 ) | | DOWN | LPE(20:5) | | UP |
|  |  | CL(74:8 ) | | DOWN | LPE(22:4) | | UP |
|  |  | CL(72:9 ) | | DOWN | LPE(22:5) | | UP |
|  |  | CL(76:13 ) | | DOWN | LPE(22:6) | | UP |
|  |  |  | |  | LPI(18:0) | | UP |
|  |  |  | |  | PC(p36:2) | | UP |
|  |  |  | |  | PE(36:6) | | UP |
|  |  |  | |  | PG(40:6) | | UP |
|  |  |  | |  | PI(34:1) | | UP |
|  |  |  | |  | PI(34:2) | | UP |
|  |  |  | |  | PI(36:1) | | UP |
|  |  |  | |  | PI(36:2) | | UP |
|  |  |  | |  | PI(36:4) | | UP |
|  |  |  | |  | PI(38:3) | | UP |
|  |  |  | |  | PI(38:4) | | UP |
|  |  |  | |  | PI(38:5) | | UP |
|  |  |  | |  | PI(40:4) | | UP |
|  |  |  | |  | PI(40:5) | | UP |
|  |  |  | |  | PS(40:6) | | UP |
|  |  |  | |  | PS(42:7) | | UP |

Supplementary Table S 4:Relative ammount( %) of each phsopholipid calls obatined fterTLC analysis and phosphorous quantification.

|  | **CTsed** | **BBNsed** | **CTex** | **BBNex** |
| --- | --- | --- | --- | --- |
| **LPC** | 3.20 ±0.26 | 4.29 ± 0.35 | 2.31 ± 0.28 | 3.01 ± 0.16 |
| **SM** | 6.00 ± 0.57 | 5.81 ± 0.64 | 5.13 ± 0.36 | 5.00 ± 0.64 |
| **PC** | 40.55 ± 0.84 | 45.23 ± 0.80^*^ | 37.18 ± 1.44^*#^ | 40.98 ± 2.35^#◊^ |
| **PI** | 9.64 ± 0.39 | 6.17 ± 0.44^*^ | 7.10 ± 1.04^●^ | 7.00 ± 0.88^●^ |
| **PS** | 6.87 ± 0.83 | 8.00 ± 1.27 | 8.35 ± 0.72 | 8.60 ± 1.40 |
| **PE** | 20.27 ± 0.50 | 19.09 ± 0.97 | 24.63 ± 2.54^*#^ | 21.20 ± 1.01^◊^ |
| **PG** | 4.66 ± 0.54 | 3.60 ± 0.57 | 4.34 ± 0.35 | 4.68 ± 0.48 |
| **CL** | 8.79 ± 0.09 | 7.80 ± 0.31 | 10.95 ± 0.69^#^ | - 1. ± 0.57 |

Statistical analysis was performed using one-way analysis of variance (ANOVA) with the Bonferroni Multiple Comparison post-hoc test. Statistics were carried out using PRISM® GraphPad Software, Inc. (La Jolla, CA, USA). A value of p <0.05 was considered significant.

*Statistically diferent from CTsed (*** p<0.001)

# Statistically diferent from BBNsed (*** p<0.001)

◊ Statistically diferent from CTex (*** p<0.001)

● Statistically diferent from CTsed (* p<0.05)

Phospholipid classes from the total lipid extract were separated by thin-layer chromatography (TLC) using plates of silica gel 60 with concentration zone 2.5 × 20 cm. Prior to separation the silica plates were washed with a solution of chloroform:methanol (1:1 v/v) and dried in the hotte for 15 min. Then, plates were dried in an oven at 100 °C for 15 min. Then, 30 μg of total PL extract dissolved in CHCl3 was applied in the TLC. The spots on TLC were dried in nitrogen flow and developed in solvent mixture with chloroform/ethanol/water/triethylamine (30:35:7:35, v/v/v/v). After complete elution, and after eluent evaporation, the TLC plates were sprinkled with a primuline solution (50 μg/100 mL acetone:water, 80:20, v/v), and visualized with a UV lamp (λ = 254 nm). Identification of the different classes of PLs was carried out by comparison with phospholipid standards. For further analysis, the spots were scraped off from the plates, and the phospholipids present in each spot were quantified using the phosphorous assay. The percentage of each PL class was calculated, relating the amount of phosphorus in each TLC spot to the total amount of phosphorus in the sample, thus giving the relative abundance of each PL class.


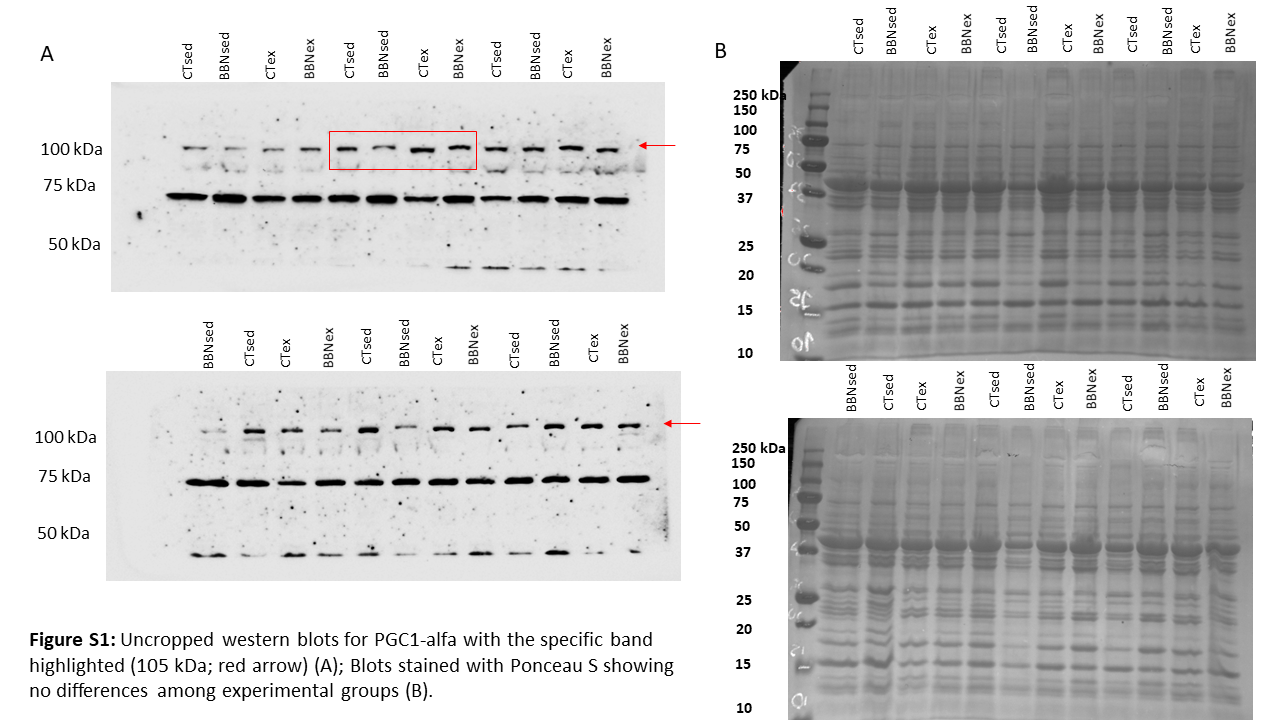


Supplementary Figure S 1: Uncropped western blots for PGC1-alfa with the specific band highlighted (105 kDa; red arrow) (A); Blots stained with Ponceau S showing no differences among experimental groups (B).

A)

B)

| **CL(72:8)** |
| --- |
|

Supplementary Figure S 2: Representative LC-ESI-MS/MS spectra of cardiolipin species, CL(72:8) as A) [M-H]^-^ ion and B) [M-2H]^2-^ ion. Informative fragments for the class are highlighted in bold and ions corresponding to the fatty acyl chains in red, all indicated in the structure scheme also shown.

| **PG(34:1)** |
| --- |
|

Supplementary Figure S 3: Representative LC-ESI-MS/MS spectrum of phosphatidylglycerol species: PG(34:1) as [M-H]^-^ ion. Informative fragments for the class are highlighted in bold and ions corresponding to the fatty acyl chains in red, all indicated in the structure scheme also shown.

| **PI(38:4)** |
| --- |
|

Supplementary Figure S 4: Representative LC-ESI-MS/MS spectrum of phosphatidylinositol species: PI(38:4) as [M-H]^-^ ion. Informative fragments for the class are highlighted in bold and ions corresponding to the fatty acyl chains in red, all indicated in the structure scheme also shown.

| **LPI(18:0)** |
| --- |
|

Supplementary Figure S 5: Representative LC-ESI-MS/MS spectrum of lysophosphatidylinositol species: LPI(18:0) as [M-H]^-^ ion. Informative fragments for the class are highlighted in bold and ions corresponding to the fatty acyl chains in red, all indicated in the structure scheme also shown.

| **PA(36:2)** |
| --- |

Supplementary Figure S 6: Representative LC-ESI-MS/MS spectrum of phosphatidic acid species: PA(36:2) as [M-H]^-^ ion. Informative fragments for the class are highlighted in bold and ions corresponding to the fatty acyl chains in red, all indicated in the structure scheme also shown.

| **PS(40:6)** |
| --- |
|

Supplementary Figure S 7: Representative LC-ESI-MS/MS spectrum of phosphatidylserine species: PS(40:6) as [M-H]^-^ ion. Informative fragments for the class are highlighted in bold and ions corresponding to the fatty acyl chains in red, all indicated in the structure scheme also shown.

| **Cer(d36:1)** |
| --- |

Supplementary Figure S 8: Representative LC-ESI-MS/MS spectrum of ceramide species: CER(d36:1) as [M+H]^+^ ion. Informative fragments for the class are highlighted in bold and ions corresponding to the fatty acyl chains in red, all indicated in the structure scheme also shown.

A) B)

| **PE(40:6)** |
| --- |
|

Supplementary Figure S 9: Representative LC-ESI-MS/MS spectra of phosphatidylethanolamine species: PE(40:6) as A)[M+H]^+^ ion and B)[M-H]^-^. Informative fragments for the class are highlighted in bold and ions corresponding to the fatty acyl chains in red, all indicated in the structure scheme also shown.

A) B)

| **LPE(20:4)** |
| --- |

Supplementary Figure S 10: Representative LC-ESI-MS/MS spectra of lysophosphatidylethanolamine species: LPE(20:4) as A)[M+H]^+^ ion and B)[M-H]^-^. Informative fragments for the class are highlighted in bold and ions corresponding to the fatty acyl chains in red, all indicated in the structure scheme also shown.

A) B)

| **PC(38:4)** |
| --- |
|

Supplementary Figure S 11: Representative LC-ESI-MS/MS spectra of phosphatidylcholine species: PC(38:4) as A)[M+H]^+^ ion and B)[M-AcO]^-^. Informative fragments for the class are highlighted in bold and ions corresponding to the fatty acyl chains in red, all indicated in the structure scheme also shown.

A) B)

| **SM(d36:1)** |
| --- |
|

Supplementary Figure S 12: Representative LC-ESI-MS/MS spectra of sphyngomyeline species: SM(d36:1) as A)[M+H]^+^ ion and B)[M-AcO]^-^. Informative fragments for the class are highlighted in bold and ions corresponding to the fatty acyl chains in red, all indicated in the structure scheme also shown.

A) B)

| **LPC(16:0)** |
| --- |
|

Supplementary Figure S 13: Representative LC-ESI-MS/MS spectra of lyosphosphatidylcholine species: LPC(16:0) as A)[M+H]+ ion and B)[M-AcO]-. Informative fragments for the class are highlighted in bold and ions corresponding to the fatty acyl chains in red, all indicated in the structure scheme also shown.
